# Programmable ROS‐Mediated Cancer Therapy via Magneto‐Inductions

**DOI:** 10.1002/advs.201902933

**Published:** 2020-05-09

**Authors:** Jiaojiao Wu, Peng Ning, Rui Gao, Qishuai Feng, Yajing Shen, Yifan Zhang, Yingze Li, Chang Xu, Yao Qin, Gustavo R. Plaza, Qianwen Bai, Xing Fan, Zhenguang Li, Yu Han, Maciej S. Lesniak, Haiming Fan, Yu Cheng

**Affiliations:** ^1^ Institute for Regenerative Medicine, Institute for Translational Nanomedicine, Shanghai East Hospital Tongji University School of Medicine 1800 Yuntai Road Shanghai 200123 China; ^2^ Collaborative Innovation Center for Brain Science Tongji University Shanghai 200092 China; ^3^ Institute for Regenerative Medicine, Institute for Translational Nanomedicine, Shanghai East Hospital Tongji University School of Medicine 1800 Yuntai Road Shanghai 200123 China; ^4^ College of Chemistry and Materials Science Northwest University Xi'an 710127 China; ^5^ Center for Biomedical Technology Universidad Politécnica de Madrid Pozuelo de Alarcón 28223 Spain; ^6^ Feinberg School of Medicine Northwestern University 676 North Saint Clair Street, Suite 2210 Chicago IL 60611 USA

**Keywords:** cancer treatment, magnetic fields, magnetic nanoparticles, reactive oxygen species, synergistic effects

## Abstract

Reactive oxygen species (ROS), a group of oxygen derived radicals and derivatives, can induce cancer cell death via elevated oxidative stress. A spatiotemporal approach with safe and deep‐tissue penetration capabilities to elevate the intracellular ROS level is highly desirable for precise cancer treatment. Here, a mechanical‐thermal induction therapy (MTIT) strategy is developed for a programmable increase of ROS levels in cancer cells via assembly of magnetic nanocubes integrated with alternating magnetic fields. The magneto‐based mechanical and thermal stimuli can disrupt the lysosomes, which sequentially induce the dysfunction of mitochondria. Importantly, intracellular ROS concentrations are responsive to the magneto‐triggers and play a key role for synergistic cancer treatment. In vivo experiments reveal the effectiveness of MTIT for efficient eradication of glioma and breast cancer. By remote control of the force and heat using magnetic nanocubes, MTIT is a promising physical approach to trigger the biochemical responses for precise cancer treatment.

## Graphical abstract

A magnetomechanical thermal induction therapy (MTIT) strategy is developed based on a single platform of assembly of magnetic nanocubes coupled with alternating magnetic fields. There is a synergistic effect between mechanical responses and thermal properties, which is highly efficient both in vitro and in vivo. The mechanisms of the synergistic effect are investigated, which can be attributed to the manipulation of biochemical reactive oxygen species (ROS) signal level. This MTIT strategy holds potential for precise treatment of deep‐seated tumors in a spatiotemporal controllability.

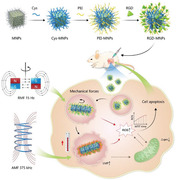

## 1 Introduction

Cancer is considered to be the leading cause of death with rapidly growing incidence and mortality worldwide.^[^ ^1^ ^]^ Multiple spatiotemporal therapeutic approaches were developed through regulation of biochemical signal transduction via physical stimuli.^[^ ^2^, ^3^, ^4^ ^]^ Reactive oxygen species (ROS) are considered as chemically reactive molecules participating in cellular signaling pathways, such as cell proliferation, differentiation, and death signaling.^[^ ^5^, ^6^ ^]^ Certain levels of ROS are required to regulate biological functions, and higher or lower levels can lead to cytotoxicity in cancer cells.^[^ ^7^, ^8^, ^9^, ^10^ ^]^

Massive ROS‐mediated anticancer strategies were mostly designed for preferentially and selectively targeting cancer treatment due to the different redox states of cancer cells and normal cells.^[^ ^11^ ^]^ Several approaches are developed to directly or indirectly increase ROS production. Partial of the chemotherapeutic drugs and agents can induce ROS generation and directly damage cell membranes or DNA, which are limited by lower delivery efficiency and a long‐term chemotherapy resistance. Enzymatic catalysis to generate ROS is highly efficient but difficult to control.^[^ ^12^ ^]^ To improve the spatiotemporal control of the ROS generation, physical stimuli are introduced to interface with biological systems in a quantitative manner. However, radiotherapy currently has to solve the side effects on the normal cells,^[^ ^13^, ^14^, ^15^ ^]^ and photodynamic therapy is limited by penetration depth of light.^[^ ^16^, ^17^ ^]^ Therefore, it is urgent to seek novel and safe physical cues to spatiotemporally regulate intracellular ROS signals, which should not be restricted by deep tissue penetration limit.

The magnetic field with remote spatiotemporal controllability and excellent tissue penetration is of great interest since it can generate a broad range of stimuli, such as mechanical force and heat,^[^ ^18^, ^19^ ^]^ in conjunction with magnetic nanoparticles (MNPs) to influence signal transduction and trigger other biological events.^[^ ^20^, ^21^, ^22^, ^23^, ^24^ ^]^ Magnetothermal properties can activate ROS generation for cancer therapy. However, the knowledge about how magnetomechanical force (MF) could induce ROS production and the crosstalk between dual physical stimuli input and biochemical signal output are limited. The force and heat generation efficiency can be amplified via assembly of magnetic nanomaterials. Under the external magnetic field, MNPs could spontaneously assemble into ordered structures with the lowest magnetic energy due to the interparticle dipolar interactions.^[^ ^25^, ^26^ ^]^ After assembly, the anisotropy constant of MNPs will be increased to elevate the magnetization of materials and enhance the magnetic responses.^[^ ^27^ ^]^ Under the magnetic field with low frequencies, ranging from 0.1‐20 Hz, MNPs can assemble and generate pN magnetomechanical forces to regulate the biochemical signaling pathways.^[^ ^28^, ^29^, ^30^, ^31^ ^]^ When exposed to the high frequency alternating magnetic field (AMF), ranging from 20–500 kHz, the aligned magnetic assemblies can convert the energy into heat in a more efficient way, which can be used in magnetic hyperthermia (MH).^[^ ^32^ ^]^

**Scheme 1:**
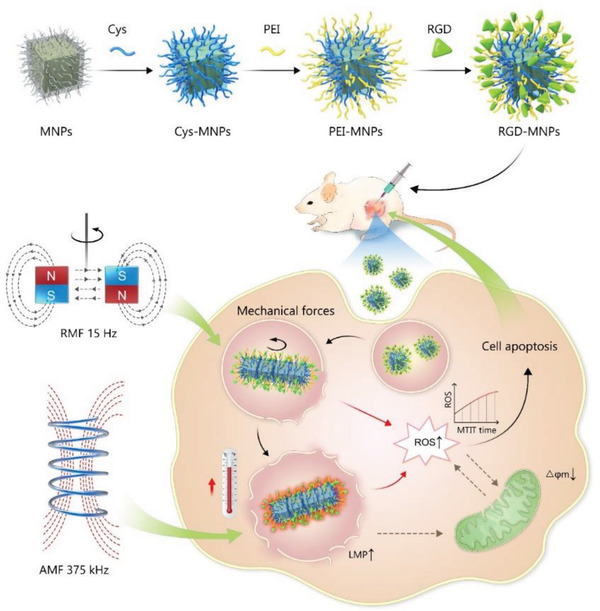
Magneto‐mechanical thermal induction therapy (MTIT) based on the assembly of nanocubes and alternating magnetic fields. The nanocubes were modified to obtain RGD functionalized nanocubes (RGD‐IONs) with cancer and neovasculature targeting ability. After internalized into lysosomes, MTIT treatment was carried out. Under 15 Hz rotating magnetic field, MF generated from assembled nanocubes rotation impaired the lysosomal membrane permeability and elevated the intracellular ROS, making cells sensitive to the subsequent heating. MH under 375 kHz alternating magnetic field further physically destroyed the lysosomes structure, induced the depolarization of mitochondria and changed the biological ROS level, leading to cell death. There was a synergistic effect between MF and MH, achieving highly efficient therapeutic effect both in vitro and in vivo.

The assembly of MNPs offers an effective approach to enhance the energy conversion efficiency into forces and heat, which could be joined together for synergistic cancer treatment. Here, we described a dual‐functional therapeutic strategy as shown in **Scheme** **1**, named mechanical‐thermal induction therapy (MTIT), based on the cooperation of two modes of the magnetic fields and assembly of RGD modified zinc‐doped iron oxide nanocubes (RGD‐IONs). As transducers for the magnetic field, RGD‐IONs could be assembled into lineage structures and convert the magnetic field energy into mechanical or thermal energy efficiently. Under 15 Hz rotating magnetic field (RMF), RGD‐IONs could assemble to form linear aggregates and generate pN mechanical forces interacting with cellular organelles and generating ROS to sensitize cancer cells. With a subsequent 375 kHz AMF treatment, assembled RGD‐IONs could further generate moderate heat to damage the sensitized cancer cells. In this MTIT strategy, cancer cells underwent apoptosis via the synergistic effect of forces and heat. The anticancer effects were further explored on two animal models.

## 2 Results and Discussion

### 2.1 Design and Characterization of Nanocubes

**Figure 1:**
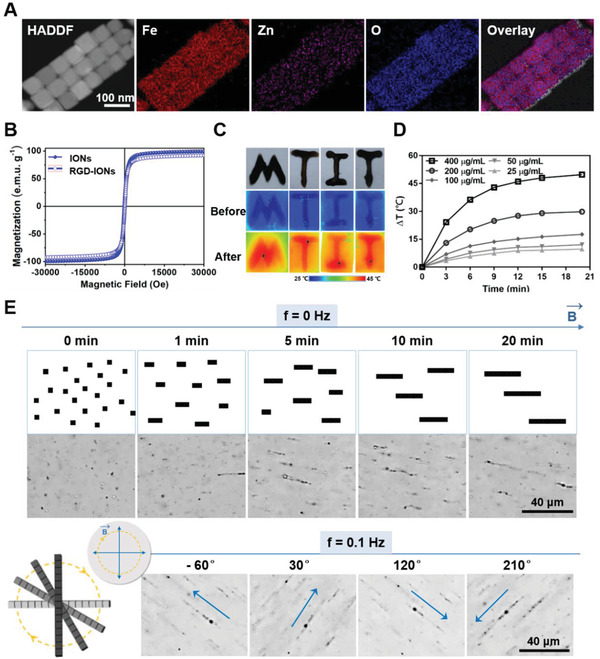
Characterization of the IONs’ physical properties. A) Elemental mapping analysis showing the composition and element distribution of IONs. B) Magnetic properties represent by M–H curves of IONs and RGD‐IONs (temperature: 298 K). C) Infrared thermal images with letter‐shaped acrylamide gel coating RGD‐IONs under AMF with frequency of 375 kHz for 20 min. D) Temperature raised curves of RGD‐IONs with different iron concentrations dispersed in water under AMF with frequency of 375 kHz for 20 min recorded by IR thermometer. E) Optical images showing the assembly of RGD‐IONs and rotation under RMF with strength of 40 mT and frequency of 0.1 Hz (the scare bar: 40 µm).

The properties of MNPs determine the magnetic responses and selectivity to cancer cells.^[^ ^33^ ^]^ The zinc‐doped iron oxide nanocubes (IONs) were synthesized via the organic thermal decomposition method and functionalized with RGD peptide to target the integrins overexpressed on the cancer cells and tumor vasculatures.^[^ ^34^, ^35^ ^]^ The shape of IONs was determined via high resolution transmission electron microscopy (HRTEM), being the average size 60 nm (**Figure** **1**A). The composition of the nanocubes was Zn_0.4_Fe_2.6_O_4_ characterized and quantified by elemental mapping analysis and energy‐dispersive X‐ray spectroscopy (EDS) as shown in Figure S1 (Supporting Information). The doping of Zn yielded an optimum saturation magnetization, reaching 98 emu g^−1^ (Figure 1B).^[^ ^36^ ^]^ These IONs were firstly modified with cysteine molecules to obtain the water solubility and further functionalized with polyethyleneimine (PEI) molecules (Figure S1, Supporting Information). The quantitative analysis for PEI modification by using nihydrin colorimetry showed that a single nanocube was covered by 3800 ± 500 PEI molecules.^[^ ^37^ ^]^ Finally, RGD peptides were conjugated on the nanocubes in order to promote cellular uptake and tumor targeting. 630 ± 30 RGD peptides per nanocube were quantified via the bicinchoninic acid (BCA) assay kit.^[^ ^38^ ^]^ The size and zeta potential changes of all intermediate products during the whole synthetic process were shown in Figure S1 (Supporting Information), which confirmed the successful modification of RGD‐IONs.

Then, the assembly, mobility, and heat generation ability of RGD‐IONs were evaluated to investigate whether RGD‐IONs could act as an effective receptor of the magnetic field. Under 0.1 Hz RMF, RGD‐IONs aligned and then rotated synchronously with the magnetic field (Figure 1C and Video S1, Supporting Information). The force was estimated via a chain model composed of assembled nanocubes.^[^ ^30^ ^]^ The magnitude of forces from an aggregate with five nanocubes was estimated to be ≈20 pN (Figure S2, Supporting Information), which was sufficient to manipulate the biochemical pathways correlated with mechanical signals.^[^ ^30^ ^]^ In addition, RGD‐IONs could efficiently convert the 375 kHz AMF energy into heat (Figure 1C,D). They had an intrinsic high specific absorption rate (SAR) value of 861.1 W g^−1^ (Figure S3, Supporting Information), implying the ability of highly efficient therapeutic effect in MH. Therefore, RGD‐IONs showed good responses to both modes of magnetic fields due to its high saturation magnetization and efficient assembly, which could generate mechanical forces and heat efficiently under RMF and AMF respectively in a spatiotemporal controlled manner. We expect that the MTIT strategy of utilizing dual physical responses via MNPs would make a breakthrough in biomedical applications, especially for the deep‐seated tumor treatment.

### 2.2 The In Vitro Anticancer Effect of MTIT

**Figure 2:**
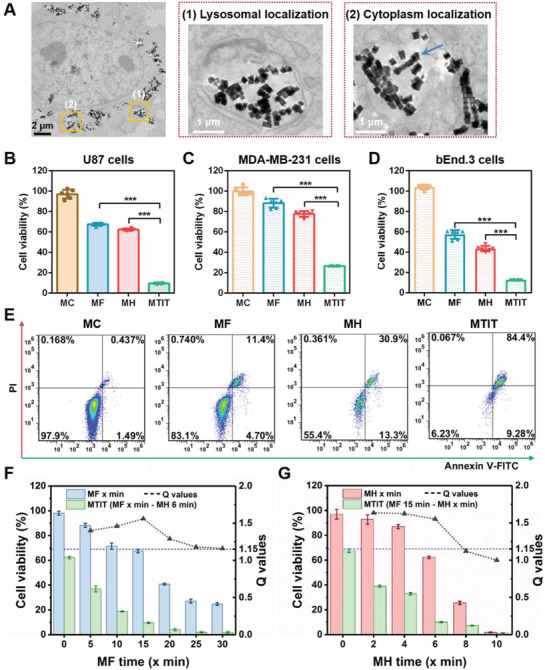
The synergistic effect of MTIT to cancer cells. A) Bio‐TEM images showed the localization of RGD‐IONs within U87 cells. RGD‐IONs were mainly localized in lysosomes and the cell cytoplasm (the black scale bar: 2 µm; the black scale bar: 1 µm). B–D) Therapeutic effects of MTIT based on RGD‐IONs to U87, MDA‐MB‐231, and bEnd.3 cells using CCK‐8 assay. The treatment parameters of MTIT were MF with 15 Hz, 15 min and MH 375 kHz, 6 min. Statistical analysis was performed using t‐test with *** indicating *p* < 0.001, ** indicating *p* < 0.01 and * indicating *p* < 0.05 (*n* = 6 per group). E) Annexin V‐FITC/PI analysis of the cells treated with materials control (MC), MF, MH, or MTIT via flow cytometry. F) MTIT with various MF treatment periods and MH 6 min. Cell viability was quantified via CCK‐8 assay. *Q* ≥ 1.15 represents the synergistic effect occurred. G) MTIT with MF 15 min and varied MH time. Cell viability was quantified by CCK‐8 assay. All these in vitro results were obtained after 24 h of application of treatment. Error bars represent standard deviations in all the plots.

In order to confirm the applicability of the MTIT strategy using RGD‐IONs for cancer treatment, the intrinsic cytotoxicity of RGD‐IONs was investigated. Over 85% of cell incubated with RGD‐IONs was found within three days of coincubation with U87 cells (Figure S4, Supporting Information). Based on the good biocompatibility, further MTIT was investigated in vitro. As shown in the bio‐TEM image (**Figure** **2**A and Figure S5, Supporting Information), RGD‐IONs were mainly located in lysosomes and partially in the cytoplasm after 24 h uptake by cells. The localization of MNPs was consistent with the literatures that the majority of the receptor‐mediated cellular uptake of nanoparticles (10–300 nm) occurred through endocytosis.^[^ ^30^, ^39^ ^]^ It was revealed that RGD‐IONs could be easily taken up by cells, suggesting that the interaction between RGD peptides and integrin receptors was beneficial. The presence of nanocubes in the cytoplasm evidenced the lysosomal escape of RGD‐IONs, which plausibly could be driven by the “proton sponge effect” of PEI molecules.^[^ ^40^ ^]^

The in vitro effect of MTIT for anticancer treatment was explored. The internalized RGD‐IONs were increased over incubation time within 24 h in U87 cells. After 24 h coincubation, there were 5.52 × 10^5^ MNPs in a U87 cell (Figure S4D, Supporting Information). For MF group, the U87 cells were treated with RMF for 15 min with the strength of 40 mT and the rotation frequency of 15 Hz. For the MH group, U87 cells were treated with AMF for 6 min with the strength of 12 kA m^−1^ (150 Oe) and the frequency of 375 kHz. For MTIT group, the U87 cells with internalized RGD‐IONs were treated with RMF and AMF orderly, with the identical parameters as above. Compared with cell viability after individual MF (67.09%) or MH (62.33%), MTIT had the lowest cell viability of 9.64% (Figure 2B). The *Q* value of MTIT was 1.56, higher than the determined threshold for the synergistic effect index of 1.15.^[^ ^41^ ^]^ It showed that the synergistic effect of MF and MH could be achieved via MTIT for U87 cells. The synergistic effect of MTIT was also observed on MDA‐MB‐231 cells (Figure 2C). Besides, MTIT in the vascular endothelial cell bEnd.3 also showed the synergistic effect of MF and MH (Figure 2D), mainly due to internalization of RGD‐IONs by the cells overexpressing *α*v integrins.^[^ ^42^, ^43^, ^44^ ^]^ It suggested that MTIT could destruct the microvascular endothelial cells at the tumor area. The corresponding live/dead cell staining was shown (Figure S6, Supporting Information), demonstrating that MTIT treatment had the largest proportion of dead cells. Cell death of synergistic MTIT was further determined by annexin V‐fluorescein isothiocyanate (FITC)/propidium iodide (PI) staining via flow cytometry (Figure 2E). Annexin V can bind to phosphatidylcholine (PS) that is transferred from inside to outside the plasma membrane at the early apoptotic process.^[^ ^45^, ^46^ ^]^ PI was used to evaluate the integrity of cell membrane, indicating late apoptosis or necrosis. 84.4% of cancer cells underwent cell apoptosis after MTIT, which was more destructive than the conventional MF (32.6%) or MH (37.7%).

To explore the role of size and shape of magnetic nanomaterials in MTIT, 22 nm magnetic nanocubes modified with RGD (RGD‐MNPs) were chosen to execute the MTIT process (Figure S7, Supporting Information). 22 nm RGD‐MNPs had a similar zeta potential (≈20 mV) (Figure S7F, Supporting Information) with the 60 nm RGD‐IONs. The saturation magnetization of 22 nm MNPs was 75 emu g^−1^ (Figure S7G, Supporting Information), lower than that of 60 nm IONs of 98 emu g^−1^, which was consistent with the literature reported showing the nanoscale size‐dependent magnetism.^[^ ^47^ ^]^ The 22 nm cubic MNPs also held the ability to align and rotate synchronously with the magnetic field (Figure S7H, Supporting Information), which could be attributed to the interparticle dipolar interactions and stabilization for assemblies,^[^ ^25^ ^]^ demonstrating the decisive role of cubic shape in the assembly. In addition, the 22 nm MNPs owned a good magnetothermal effects (Figure S7I, Supporting Information) attributed to its zinc‐doped composition and anisotropy.^[^ ^36^ ^]^ The synergistic effect of MTIT utilizing 22 nm RGD‐MNPs was observed (Figure S8D, Supporting Information). However, the MTIT anticancer effect mediated by 22 nm RGD‐MNPs was ≈39%, which was 2.3 times weaker than that of 60 nm RGD‐IONs (≈90%) with the similar internalized iron amount (Figure S9, Supporting Information). It suggested that the size of nanoparticles was an important factor in the magnetically induced mechanical and thermal effect. From the theoretical calculation of forces (Figure S8E, Supporting Information) and experimental data of SAR value (Figure S8F, Supporting Information), it could be concluded that the moderate therapeutic effect of MTIT mediated by 22 nm RGD‐MNPs was related to the size‐dependent properties, such as magnetism, assembly, forces and heat generation.

To further explore the role of assembly in MTIT, ferumoxytol, U. S. Food and Drug Administration (FDA)‐approved 13 nm iron oxide nanoparticles for the treatment of iron deficiency anemia,^[^ ^48^ ^]^ were chosen to execute the MTIT process (Figure S10, Supporting Information). It could not form the linear aggregates under the same condition (Figure S10C, Supporting Information) because nanospheres had a much smaller contact area and weaker attractive interactions, providing no stabilization for assemblies.^[^ ^25^ ^]^ It demonstrated that the shape of nanoparticles played a decisive role in the magnetic field‐induced assembly. The SAR value of ferumoxytol was 76.3 W g^−1^ (Figure S10F, Supporting Information), demonstrating its low magneto‐thermal property. The cell viability was 64.6%, 94.7%, and 69.1% for MTIT, MF, and MH group, respectively. No synergistic effect of MTIT was detected (Figure S10H, Supporting Information). The minor difference between MTIT and MH group was mainly due to the low assembly efficiency (Figure S10C, Supporting Information), which could not enhance the MH effect. The weak dipolar interaction between ferumoxytol nanoparticles with good dispersity showed negligible effects to enhance mechanical forces.^[^ ^25^ ^]^ This phenomenon indirectly proved the key role of MF dependent on the assembly, synergistically contributing to cell death.

To fully investigate the MTIT effect to normal cells, astrocytes and 3T3 fibroblast cells were chosen to evaluate the selectivity of RGD‐IONs and cytotoxicity of MTIT. Astrocytes were the most abundant cell type of the neuroglia in mammalian brain and have several crucial functions, e.g., regulated synaptic transmission and neuronal excitability, and was involved in creating and maintaining the blood‐brain barrier.^[^ ^49^ ^]^ As shown in Figure S11A (Supporting Information), RGD‐MNPs were internalized into astrocytes due to the presence of *α*v*β*3 on the surface.^[^ ^50^ ^]^ There was a decrease of astrocytes viability under AMF coupled with RGD‐IONs groups (Figure S11B, Supporting Information), which was consistent with the literature elaborating that astrocyte cell membrane structure could be damaged when exposed to AMF and MNPs.^[^ ^51^ ^]^ By comparing the cell viability of astrocytes and U87 cells after MTIT treatment, lower cytotoxicity was achieved in astrocytes, suggesting the cancer cells were more sensitive to MTIT. In addition, the cytotoxicity of MTIT on normal 3T3 fibroblast cells was lower than that on U87 cells in accordance with the cellular uptake of RGD‐IONs (Figure S11C, Supporting Information).

To investigate the synergistic effect, the MF and MH doses were optimized in the MTIT. Considering of the effective heating with the shortest MH time, we selected the treatment time of 6 min for MH procedure, resulting in a temperature below 44 °C (Figure S12, Supporting Information). MTIT groups were performed with various MF time, 5, 10, 15, 20, 25, and 30 min and a fixed MH time 6 min (Figure 2F). The calculated Q values were 1.40, 1.46, 1.56, 1.29, 1.18, and 1.16 respectively, higher than the determined threshold for the synergistic effect index of 1.15.^[^ ^41^ ^]^ For the MTIT with various MF treatment periods and MH 6 min, the synergistic effect index reached the maximum when MF lasted for 15 min (Table S1, Supporting Information). Then, the MH dose was further optimized (Figure 2G), in which the MTIT groups had fixed MF treatment (15 min) and various MH treatment periods including 2, 4, 6, 8, 10 min. The calculated *Q* values were 1.64, 1.62, 1.55, 1.12, and 1.00, respectively. One important phenomenon to note was that the *Q* value was approximately equal to 1 over MH 8 min due to the relative high MH treatment temperature (over 44 °C) to kill cancer cells (Table S2, Supporting Information). Thus, treatment times were set to the optimized values of 15 and 6 min for MF and MH respectively for the subsequent experiments. Both sets of experiments exhibited widely synergistic effects, verifying that there was an inherent crosstalk between MF and MH.

The biological effects of magnetic fields should also be taken into consideration (Figure S13, Supporting Information). No significant cytotoxicity was observed in the cells treated with RMF only, suggesting the important role of RGD‐IONs for magnetomechanical destruction. The AMF treated cells could slightly decrease the average cell viability due to the existence of dipolar polarization.^[^ ^52^, ^53^ ^]^ For MTIT group, the cell viability had a bit decrease but no synergistic effect was observed. The inhibitory effect was influenced by the mode, intensity, frequency and treated time of magnetic field, as well as the dose of nanomaterials. It should be also noted that magnetic field could inhibit or stimulate cancer cells which varies on cell types and exposure conditions.^[^ ^54^, ^55^, ^56^, ^57^, ^58^ ^]^

### 2.3 Mechanisms of MTIT for Cancer Destruction

**Figure 3:**
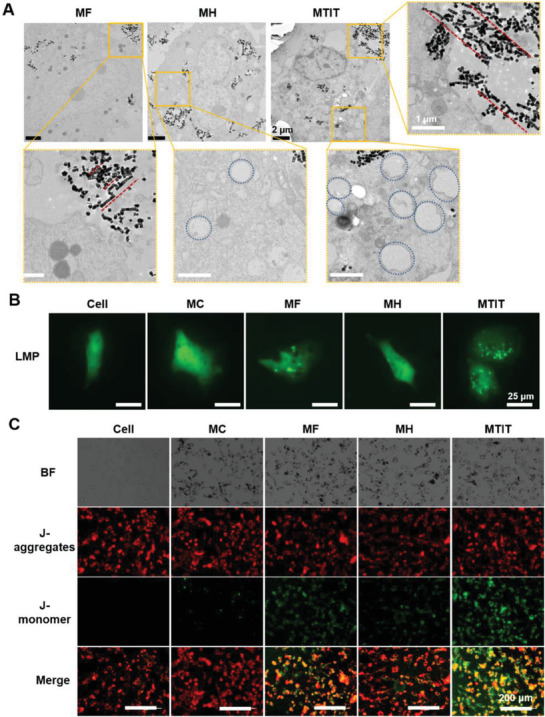
Cell structure destruction via MTIT. A) Cell structure changed after different treatments observed by bio‐TEM. Red lines showed the alignment of nanocubes and the blue circles represented the cytoplasmic vacuolization. Scale bar in zoomed picture, 1 µm. B) Lysosomal membrane permeability characterized by eGFP‐Gal3 plasmid. Representative images showed that the number of fluorescent spots increased indicating the LMP increase. Scale bar: 25 µm. C) Mitochondrial membrane potential imaging by JC‐1 staining. The images show normal mitochondria with polarized membrane (red fluorescence of J‐aggregates) and damaged mitochondria with depolarized membrane (green fluorescence of J‐monomer). Scale bar: 200 µm.

To further investigate the mechanisms of the synergistic effect of MTIT, the primary physical cellular structure destruction and secondary biochemical signals were studied. First, the effects of MTIT on cellular structures were observed and compared with other treatment groups. Cells in MTIT group turned rounded after 1 h following the treatment, while in MH group changes were visually apparent after 4 h and cells in the MF group showed minor changes in cell morphology (Figure S14, Supporting Information). These changes demonstrated that MF effect could trigger cells' susceptibility to heat. As observed in bio‐TEM (**Figure** **3**A) images, RGD‐IONs could aggregate to chains by MF under RMF treatment. The alignment was also found in MTIT (Figure S15, Supporting Information).

Considering that the packing of internalized RGD‐IONs caused the clustering effect that may lead to enhanced magnetic properties, it was necessary to differentiate “intracellular” magnetic field induced alignment from intracellular “packing or clustering.” In the presence of magnetic field, the magnetic assemblies were longer and more orderly than those in the absence of magnetic stimulation (Figure S15, Supporting Information and Figure 2A). Without the magnetic field, the dipoles were randomly oriented and the weak dipole‐dipole interactions were usually not sufficient to result in the chain formation. When the nanocubes contact distance was decreased, van der Waals interactions would play a role between the adjacent nanocubes,^[^ ^59^ ^]^ which may induce the formation of chain‐like structures to some degree. In contrast, when an external magnetic field was applied, MNPs tended to align along the magnetic field and assembled into organized anisotropic structures and patterns due to magnetic dipole‐dipole interactions.^[^ ^25^ ^]^ It should be noted that the anisotropy associated 1D assemblies’ magnetism was enhanced with higher coercivity compared to the randomly aggregated nanoparticles,^[^ ^27^ ^]^ which led to increased magnetic properties for the improved therapeutic effects.^[^ ^32^, ^60^ ^]^ In MTIT group, cancer cell shrinking and blebbing were observed, which were features of the programmed cell death consistent with the results described above.^[^ ^61^ ^]^ An interesting phenomenon to be noted was that cells in MH and MTIT appeared to undergo cytoplasmic vacuolization, which was a sign of paraptosis.^[^ ^62^ ^]^

Then, the subcellular structure, lysosomal membrane permeabilization (LMP), was evaluated by enhanced green fluorescent protein (eGFP)‐modified Gal‐3 plasmid.^[^ ^63^ ^]^ When the LMP increased, plasmids gather in lysosomes and form fluorescent spots. Most fluorescent spots were observed in MTIT group, indicating its great effect on inducing LMP (Figure 3B). There were a few fluorescent spots in cells treated with MF and MH, showing that individual MF or MH treatment had ability to destroy the lysosome membrane directly or indirectly via mechanical forces or heat. The amplitude of the destruction could be quantified as a function of the treatment time (Figure S16B, Supporting Information), showing that the LMP had a positive correlation with the treatment time. Compared with MF and MH under the same duration for each treatment, the number of fluorescent spots after MTIT was about three times higher compared to MF, representing the strongest LMP effect. Besides, the normalized mean fluorescent intensity of lyso‐traker red, which relies on the acidic substance in the lysosomes, was determined. Cells in the MTIT group showed 41% of the fluorescence intensity when compared to the control group, which was significantly lower than the MF or MH group (Figure S11, Supporting Information). It further indicated the MTIT showed the best effect to destruct lysosomes.

The increased LMP can induce the mitochondrial dysfunction based on the lysosomal‐mitochondrial axis theory,^[^ ^64^, ^65^ ^]^ elaborating that cathepsins leaked from the lysosomes could interact with mitochondria and induce the decrease of mitochondrial membrane potential (MMP), although few MNPs were directly bounded to the mitochondrial membrane (Figure S17, Supporting Information). JC‐1 dye, with a potential‐dependent accumulation property, was widely used to estimate the MMP.^[^ ^66^ ^]^ When the MMP decreased, the JC‐1 would translocate from mitochondria to cytoplasm, with the fluorescence emission from red to green, representing J‐aggregates and J‐monomer, respectively. Therefore, mitochondria depolarization can be imaged by the increased ratio of green/red fluorescent intensity. The ratio of green/red intensity in the MTIT group was quantified as 0.59, which was three to five times higher than that in MF and MH, respectively (Figure 3C and Figure S16E, Supporting Information). All these results showed that MTIT produced the strongest destruction on cancer cell structures, especially for lysosomes and mitochondria, characterized by increased LMP and decreased MMP, as well as cytoskeleton disruption (Figure S18, Supporting Information).

**Figure 4:**
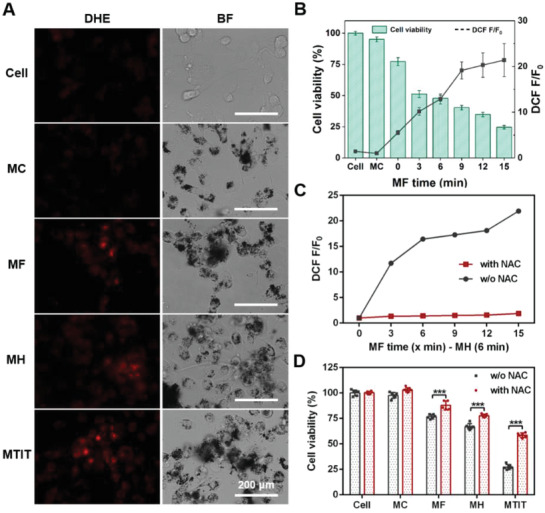
MTIT Programmed generation of ROS to manipulate cancer cell viability. A) Generation of superoxide anion observed by DHE staining under live cell imaging system. The bright field (BF) images were also shown. Scale bar: 200 µm. B) Programmed generation of ROS controlling the cell viability in MTIT groups with varied MF treated time. Intracellular ROS level was determined by DCFH‐DA staining and detection by flow cytometry. Cell viability was measured by CCK‐8 assay kit post treatment 24 h. C) The scavenging effect of NAC. NAC, a scavenger of ROS, could be used to eliminate the intracellular ROS level. The ROS level was reflected by DCF fluorescence. DCF F represent the intracellular ROS level after treatment, while DCF F0 represent the initial ROS level before treatment. D) Cell viability increased with the decrease of ROS. The U87 cells were pretreated with or without NAC for 2 h, followed by the different treatments. Cell viability increased with the addition of NAC to some extent. Statistical analysis was performed using t‐test with *** indicating *p* < 0.001, ** indicating *p* < 0.01 and * indicating *p* < 0.05 (*n* = 6 per group). Error bars represent standard deviation.

In addition to cellular structure changes, the secondary biochemical signals may also be regulated by the physical stimuli. It is well known that ROS are abundant in lysosomes and mitochondria.^[^ ^67^, ^68^ ^]^ We hypothesized that the intracellular ROS would be elevated due to the increased LMP and decreased MMP, according to the lysosomal‐mitochondria axis theory proposed in the literatures.^[^ ^65^, ^69^ ^]^ Dihydroethidium (DHE) probe was used to study the intracellular superoxide anion, ROS were produced partially in mitochondrial respiratory chain.^[^ ^70^ ^]^ The red fluorescent intensity of DHE was apparent in treated groups, especially in MF and MTIT (**Figure** **4**A). The quantitative analysis of percentage of DHE positive cells was shown (Figure S19A, Supporting Information). It could be observed a high level of superoxide anions in MF and MTIT groups, with the proportion of DHE positive cells more than 30%. In contrast, the proportion of DHE positive cells in the control group was less than 3%. Similar results were obtained via 2,7‐dichlorodi‐hydrofluorescein diacetate (DCFH‐DA) for detecting the overall ROS level by using flow cytometry (Figure S19B, Supporting Information). It indicated that mechanical stimuli could effectively up‐regulate the biochemical ROS signals, which could explain the sensitizing of cells by MF for the subsequent MH. In order to prove that ROS played a key role for the synergistic effect, ROS content of MTIT with varied MF time and fixed MH time of 6 min were quantified by flow cytometry. It could be observed that intracellular ROS increased over MF treatment time, implying that MF treatment could programmatically regulate the intracellular ROS content (Figure 4B). In addition, cell viability was decreased over treatment time. As expected, there was no significant correlation between the ROS level and treatment duration for the MF control group (Figure S19D, Supporting Information), which may be one of the plausible explanations for the lack of synergistic effect of MTIT without MNPs. Thus, cell viability could be adjusted by the ROS concentrations, dependent on MF treatment time. The elevated ROS could mainly generate from two pathways: 1) ROS leaked from lysosomes and mitochondria due to increased LMP and decreased MMP; 2) ROS production occurred when cells were exposed to a number of proapoptotic agents which induced cathepsins released from lysosomes.^[^ ^71^ ^]^ In addition, the HSP 70 level in the cells was also affected post the MTIT treatment (Figure S19E, Supporting Information). Compared to the MH treated cells, both MF and MTIT showed the down regulation of HSP 70, suggesting other biological signaling pathways could be regulated besides ROS.

To further explore the ability of ROS to regulate cell viability, N‐acetyl‐L‐cysteine (NAC), a scavenger of ROS (Figure 4C),^[^ ^72^ ^]^ was chosen to eliminate the killing effect of ROS. As shown in Figure 4D, cell viability in MTIT pretreated with NAC increased 2.2 times from 27.2% to 58.5%, while the cell viability in MF increased 1.1 times from 76.7% and in MH increased 1.2 times from 67.0%, proving that ROS were the crucial signal molecules for the synergistic effect in MTIT. After elimination of ROS, cytotoxicity in MTIT treated cells still existed, and the probable reasons could be attributed to the direct mechanical force destruction and thermal denaturation. Thus, ROS was verified to be the key biochemical signal in the synergistic effect of MF actuation and encoding cancer cells susceptible to heat in MTIT.

### 2.4 Therapeutic Effect of MTIT In Vivo

**Figure 5:**
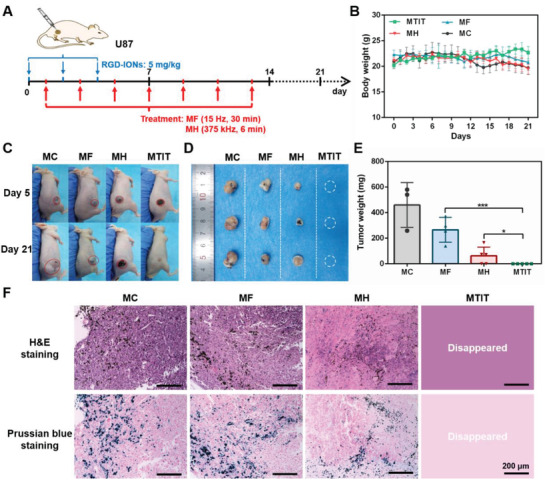
Therapeutic effect of MTIT in U87‐bearing mice. A) Schematic illustration the process of in vivo treatment. RGD‐IONs were intratumorally injected with iron 5 mg kg^−1^ per time for three times at day 0, 2, and 4. The treatments were performed for seven times, at day 1, 3, 5, 7, 9, 11, and 13, respectively. The parameters of RMF 40 mT, 15 Hz, and 30 min. The parameters of AMF were 375 kHz, 12 kA m^−1^ and 6 min. B) Body weight recorded daily for 3 weeks. C) Mice pictures at day 5 and day 21. D,E) Tumor pictures and tumor weight obtained after dissection for four groups. Statistical analysis was performed using t‐test with *** indicating *p* < 0.001, ** indicating *p* < 0.01 and * indicating *p* < 0.05. Error bars represent standard deviation. F) H&E staining and Prussian blue staining of tumor tissues dissected from the mice at day 21. Scale bar: 200 µm.

After exploring the synergistic mechanism at the cell culture level, the in vivo therapeutic effect of MTIT was investigated and carried on the U87 heterotypic mouse model. RGD‐IONs were intratumorally administrated three times at the day 0, 2, and 4 with a dose of 5 mg kg^−1^ per injection after tumor volume reached 100 mm^3^ (**Figure** **5**A). The intratumor injection combined with the spatiotemporally controlled magnetic fields could ensure the localized generation of shearing forces and/or heat in order to minimize the side effects to the normal cells. RMF and AMF were performed seven times every 2 days, from day 1 to day 13, and the treatment parameters were 40 mT, 15 Hz, 30 min for MF and 12 kA m^−1^ (150 Oe), 375 kHz, 6 min for MH, respectively. It was worth noting that the parameters of RMF and AMF in the studies were within the safety range respectively.^[^ ^19^, ^73^ ^]^

The mouse body weight and tumor size were monitored daily for 3 weeks. The body weight of mice remained stable (Figure 5B). Tumors in MTIT group were completely eliminated. MF showed a slight inhibitory effect, while MH dramatically reduced tumor size (Figure 5C,D and Figure S20, Supporting Information). The quantitative analysis of tumor weight was shown in Figure 5E, which was consistent with the tumor images. To investigate the interaction of RGD‐IONs with tumor tissues, Prussian blue was used as the chromogenic agent of nanocubes. RGD‐IONs could accumulate at the tumor area, which was promoted by RGD targeting (Figure 5F). The iron signal was also observed in the spleens (Figure S20C, Supporting Information), suggesting the possible distribution of RGD‐IONs. Combined with the hematoxylin–eosin (H&E) staining results (Figure S20D, Supporting Information), no obvious damage effect of RGD‐IONs was found in heart, liver, spleen, lung, kidney tissues posttreatments, demonstrating the safety of MTIT. It should be noted that intracranial injection of MNPs for MH was recommended in clinical studies for glioblastoma treatment to minimize the body side effects.

**Figure 6:**
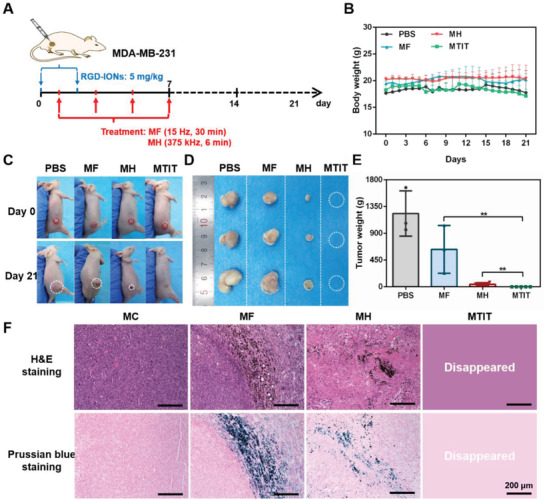
Therapeutic effect of MTIT in MDA‐MB‐231‐bearing mice. A) Schematic illustration the process of in vivo treatment. RGD‐IONs were intratumorally injected with iron 5 mg kg^−1^ per time at day 0 and 2. The treatments were performed at day 1, 3, 5, and 7, for four times. The parameters of RMF 40 mT, 15 Hz, and 30 min. The parameters of AMF were 375 kHz, 12 kA m^−1^, and 6 min. B) Body weight recorded daily for 3 weeks. C) Mice pictures at day 0 and day 21. D,E) Tumor pictures and tumor weight obtained after dissection for four groups. Statistical analysis was performed using t‐test with *** indicating *p* < 0.001, ** indicating *p* < 0.01 and * indicating *p* < 0.05. Error bars represent standard deviation. F) H&E staining and Prussian blue staining of tumor tissues dissected from the mice at day 21. Scale bar: 200 µm.

To further evaluate MTIT for cancer treatment, MDA‐MB‐231 subcutaneous model was constructed. In vitro experiments also confirmed the synergistic effect of MTIT, verifying the general applicability of this strategy. It should be noted that MDA‐MB‐231 and U87 cells had different susceptibility to heat and the treatment parameters were adjusted to the effective range accordingly. RGD‐IONs were intratumorally injected twice with a dose of 5 mg kg^−1^ each time after the tumor volume reached to 100 mm^3^. And RMF and AMF were performed four times with the same treatment parameters (**Figure** **6**A). The body weight posttreatment in different groups was monitored and was stable in control and treated groups (Figure 6B). The MF treatment had a moderate inhibitory effect when compared to the control group and MH could greatly reduce the tumor volume (Figure 6C–E and Figure S21, Supporting Information). In contrast, MTIT could completely eliminate tumors. The overall results proved that MTIT strategy could achieve great efficacies in multiple tumor models.

Moreover, the histopathological results confirmed the RGD‐IONs mainly located in the tumor area after the cancer cell death (Figure S21C, Supporting Information). During the treatment, the cancer cells membrane could be destroyed to some extent, leading to the partial release of RGD‐IONs. The reasonable possibilities regarding the destiny of RGD‐IONs released from the dead cells were investigated. Post direct physical and ROS destruction occurred in the cancer cells, RGD‐IONs could be released from the damaged cell and be taken up again by the neighbor cells (Figure S22, Supporting Information). The MTIT mediated by reinternalized RGD‐IONs could preserve the destructive effect for treating cancer cells (Figure S23, Supporting Information). It was also likely that the RGD‐IONs released from dead cells could recruit monocytes to the site of particle through local expression of chemotactic cytokines and induce a proinflammatory polarization inhibiting tumor growth.^[^ ^74^, ^75^ ^]^ According to the literature reported, macrophages could be polarized to the tumoricidal M1 type with a proinflammatory immune response when exposed to ferumoxytol nanoparticles, accompanied with the elevated ROS production due to the Fenton reaction, which would increase cancer cell apoptosis.^[^ ^76^, ^77^ ^]^ Overall, the MTIT therapeutic effect could be attributed to the direct physical destruction, indirect biochemical signals (ROS) damage, as well as activation of immune responses.

## 3 Conclusion

An effective MTIT strategy was designed to treat cancers depending on RGD‐IONs coupled with alternating magnetic fields. First, the multifunctional RGD‐IONs with excellent assembly efficiency and magnetic responses were designed as the magnetic antennas, converting magnetic field energy into forces and heat in a programmed fashion. Secondly, MTIT produced a synergistic effect to treat cancer cells which was highly efficient both in vitro and in vivo. Moreover, the mechanisms of the synergistic effect were explored covering cellular organelles and biochemical signals. The increased LMP, followed by decreased MMP and accompanied with massive production of ROS made cancer cells susceptible to the MH through mild hyperthermia. During the process, ROS generation could be programmed via destructing the subcellular structures under the magnetic field. That means of physical stimuli input can be converted to biochemical signals output, which allows manipulating the cancer cell fate in a programmed way. Since the physical stimuli in this system can be customized by the magnetic response properties of MNPs, such as saturation magnetization, and the external magnetic field parameters containing field strength, frequency, and exposed time, the biochemical signal output can be controlled in a spatial, temporal, and quantitative manner to meet various needs in biomedical applications.

In the future studies, we will focus on establishing the correlations between the magnetic‐based stimuli and the biochemical signals, as well as the immune responses. It is important to point out that other biochemical signals, such as HSPs and Ca^2+^ exchange, should be explored in depth in order to understand how cells interact with physical stimuli and guide a better design of MNPs with optimized magnetic parameters. And it is valuable to explore how to activate the immune response via the physical‐biological cues, which would be ideal for the sustained treatment or long‐term protection. Besides, we will continue to explore the interaction and influence of these two physical stimuli, providing the theoretical basis for clinical translation of the synergistic therapy. To accelerate the clinical applications of the magneto‐therapy, the administration of the nanomaterials combined with magnetic navigation and imaging guidance such as MRI or MPI will be worthy to explore. The preclinical studies on multiple animal models to supplement the metabolism of magnetic nanomaterials will be helpful to fully investigate the effectiveness and safety of the magnetostrategy.

## 4 Experimental Section

##### Materials

Iron (III) acetylacetonate (Fe(acac)_3_, 97%, Sigma‐Aldrich), zinc acetylacetonate hydrate (Zn(acac)_2_, 97%, Aladdin), oleic acid (OA, AR grade, Aladdin), dibenzyl ether (>98%, Sigma‐Aldrich), L‐cysteine (Cys, 99%, Shanghai Macklin Biochemical Co., Ltd.), 1‐(3‐dimethylaminopropyl)‐3‐ethyl carbodiimide hydrochloride (EDC, >98.0%, Aladdin), N‐hydroxysuccinimide (NHS, 98%, Aladdin), ethylene imine polymer (PEI, M. W. 10 000, 99%, Shanghai Macklin Biochemical Co., Ltd.), RGD peptide (RGDRGDRGDRGDPGCL, 98.7%, Shanghai GL Biochem Ltd.) were purchased. Ethanol (CP grade, Sinopharm Chemical Reagent Co., Ltd.), toluene (Sinopharm Chemical Reagent Co., Ltd.), agarose (BR, 90%, Sigma‐Aldrich), acrylamide (AA, Aladdin), ammonium persulfate (APS, ≥ 98%, RT, Sigma‐Aldrich), N, N‐methylenebisacrylamide bis‐acrylamide (BAA, 99%, Shanghai Macklin Biochemical Co., Ltd.), N, N,N’,N’‐ tetramethylethylenediamine (TEMED, 99%, Aladdin), hydrochloric acid (HCl, AR, Sinopharm Chemical Reagent Co., Ltd.), nitric acid (HNO_3_, AR, Sinopharm Chemical Reagent Co., Ltd.) were purchased. Nihydrin colorimetry assay kit (Beijing Leagene, Inc.), enhanced BCA protein assay kit (Beyotime Biotech, Inc.), phosphate buffer saline (PBS, Hyclone), Dulbecco's modified Eagle's medium (DMEM)/high glucose (Hyclone), penicillin/streptomycin (Hyclone), and fetal bovine serum (FBS, Hyclone) were purchased. CCK‐8 assay kit (Dojindo Molecular Technologies, Inc.), calcein‐AM (Thermo Fisher Scientific, Shanghai, China), propidium iodide (PI, Beyotime Biotech, Inc.), annexin V‐FITC/PI apoptosis detection kit (Shanghai Sangon Biotech Co., Ltd.), pCDH‐EF1‐EGFP‐LGALS3 plasmid (EGFP‐Gal_3_, B. Liu provided), Lipo6000 transfection agent (Beyotime Biotech, Inc.), lyso‐Tracker Red DND‐99 (Beyotime Biotech, Inc.), MitoProbe JC‐1 Assay Kit (Thermo Fisher Scientific, Shanghai, China), phalloidin‐iFluor 555 Conjugate (AAT Bioquest, Inc.), Hoechst33342 (Dojindo Molecular Technologies, Inc.), dihydroethidium (DHE, Beyotime Biotech, Inc.), DCFH‐DA (Beyotime Biotech, Inc.), N‐acetyl‐L‐cysteine (NAC, Beyotime Biotech, Inc.), and prussian blue assay kit (Beijing Solarbio Science & Technology Co., Ltd.) were purchased. Ultrapure water was used throughout all experiments.

##### Rotating Magnetic Field Setup

The MFG‐100 magnetic field (MagnebotiX AG, Zurich), integrated with an inverted fluorescence microscope (Olympus, Japan), was applied for in vitro video recording.

The NdFeB based RMF station was integrated with two NdFeB magnets in a rotating cylinder (Niumag Co., Ltd., Shanghai, China) for cells and in vivo experiments. At the distance of 5 mm above the station, the magnitude of RMF was 40 mT.

##### Alternating Magnetic Field Setup

The AMF (SPG‐06A‐II, Shenzhen Shuangping Power Technology Co., Ltd.) was set as frequency of 375 kHz and magnetic strength of 12 kA m^−1^ (150 Oe), which was used in in vitro and in vivo experiments. The AMF (magnetic thermal instrument‐M5, SuperMag Technology) with frequency of 399 kHz combined with fiber optic temperature converter (Photon Control) were used to measure the SAR values of RGD‐IONs.

##### Synthesis of 60 nm Zinc Doped Iron Oxide Nanocubes (IONs)

IONs were synthesized through organic solution‐phase decomposition method, which was referred to previous reports. Detailed information go as follows: Fe(acac)_3_ (0.8 mmol, 282.5 mg) and Zn(acac)_2_ (1.2 mmol, 316.3 mg) were dissolved in dibenzyl ether (52.6 mmol, 10.4 mL) and oleic acid (3.8 mmol, 1.2 mL). The mixture was sonicated 5 min to disperse, followed by degassed under argon for 30 min under 600 r min^−1^ stirring. Then it was heated to 290 °C and held 30 min for nucleation and growth under argon and stirring all the time. After cooling to room temperature, 30 mL ethanol was added into reaction system to promote the precipitation of nanocubes. Then the products were washed by ethanol and toluene three times orderly. Finally, the IONs were dispersed in ethanol.

##### Synthesis of IONs@Cys (Cys‐IONs)

To promote the biocompatibility of IONs, they were first transferred from organic phase to aqueous phase by modifying cysteine on IONs surface. 5 mg IONs dispersed in 13 mL ethanol were mixed with 25 mg cysteine dissolved in 2 mL water, followed by ultrasonic probe (Fisher Scientific 120) with power 40 W for 1 h. The set up were working time 5 s and interval time 2 s. Afterwards Cys‐IONs were washed and purified three times, achieving with good dispersity in 10 mL water.

##### Synthesis of IONs@Cys@PEI (PEI‐IONs)

To improve the stability of Cys‐IONs, PEI was selected and modified on their surface due to its high positive charged characteristic. The reaction was catalyzed by EDC and NHS. Briefly, EDC (0.1 mmol, 19.17 mg) and NHS (0.11 mmol, 12.66 mg) dissolved in 1 mL water respectively were added to above Cys‐IONs to active the carboxyl group of outer cysteine with probe ultrasonication for 0.5 h. Then 50 mg PEI dissolved in 3 mL water was added to the above mixture, maintaining ultrasonic treatment for another 1 h. Finally, PEI‐IONs were obtained, washed and purified for three times, achieving good dispersity in 10 mL water.

##### Synthesis of IONs@Cys@PEI@RGD (RGD‐IONs)

To promote the targeting ability and improve the efficacy of MNPs, RGD was functionalized on the surface of PEI‐IONs. The amidation reaction was also catalyzed with EDC and NHS. EDC (0.2 mmol, 38.34 mg) and NHS (0.22 mmol, 25.32 mg) were dissolved in 2 mL water, respectively. The ultrasonication method was used to activate the carboxyl group of RGD molecules for 30 min by mixing the RGD (2.4 mg) dissolved in 4 mL water and EDC/NHS solution. Then, PEI‐IONs were added to the reaction system and keep the ultrasonic treatment for another 1 h. Finally, RGD‐IONs were obtained, followed by washed for three times and stored in 10 mL water at 4 °C.

##### Synthesis and Modification of 22 nm Zinc‐Doped Iron Oxide Nanocubes@Cys@PEI@RGD (RGD‐MNPs)

The synthetic process of 22 nm MNPs was similar to the 60 nm IONs, referred to the same literature. During the synthesis of 22 nm MNPs, 400 mg of 4‐biphenyl‐carboxylic acid and oleic acid (3.78 mmol, 1.198 mL) was also added in the reaction system. The following purification, functionalization, and characterization were same as that of 60 nm.

##### Characterization of Morphology and Composition of IONs

The morphology and average size of IONs were characterized by high resolution transmission electron microscopy (JEOL JEM‐2100F, 200 kV) and transmission electron microscopy (TEM, JEM‐1230, JEOL Ltd.) The qualitative and quantitative analysis of the composition was using elemental mapping and energy dispersive X‐ray spectroscopy (EDS, Oxford x‐met 8000) and inductively coupled plasma‐optical emission spectroscopy (Thermo iCAP 7600 ICP‐OES). Dynamic light scattering (DLS, Zeta SIZER NANO ZS90, Malvern Ltd.) was used measure the hydrodynamic particle size and zeta potential of different MNPs (Figure S1, Supporting Information).

##### Characterization of Magnetic Properties of RGD‐IONs

The static magnetic properties of dry IONs and RGD‐IONs at room‐temperature were measured by vibrating sample magnetometer (VSM, Lakeshore 7407, US). The mechanical force generated properties were recorded by MFG‐100 setup. The RGD‐IONs were dispersed in water with the concentration of Fe 20 µg mL^−1^, putting it on the magnetic field set at 40 mT and changed with different frequencies. The magnetic heating property was measured by using AMF with frequency of 375 kHz and time of 20 min, recorded by thermal imaging system (FOTRIC). The RGD‐IONs were dispersed and fixed in the AA hydrogel, referred to the article published before. The temperature changed curves were measured with different concentration of Fe of RGD‐IONs under AMF with frequency of 375 kHz and time of 20 min, and also recorded by thermal imaging system. The SAR values measurement was used AMF (SuperMag Technology) with frequency of 399 kHz, electric current of 25 A, magnetic strength of 330 Oe, recording with fiber optic temperature converter (Photon Control). The system was RGD‐IONs dispersed in 1% agarose gel, with the final concentration of RGD‐IONs of 100 µg mL^−1^. The magnetic responses of ferumoxytol were also investigated as method described above.

##### Qualitative and Quantitative Analysis of Modified Molecules on RGD‐IONs

FTIR spectrum was used to verify the successful modification of Cys on the surface of IONs (Figure S1, Supporting Information). To quantify the amount of PEI molecule on a single Cys‐ION, nihydrin colorimetry was performed referred to the introduction. Further, BCA protein assay kit was used to quantify the RGD molecule number on a single PEI‐ION.

##### Cell Culture

U87 (American Type Culture Collection, Manassas, VA., USA) and MDA‐MB‐231 cells were cultured in high glucose DMEM medium containing 10% fetal bovine serum (FBS) and 1% penicillin/streptomycin, and then incubated at 37 °C under a humidified atmosphere containing 5% CO_2_. Astrocytes, bEnd.3 cells and 3T3 cells were cultured with changed FBS‐141 at the same incubation environment.

##### Biocompatibility of RGD‐IONs in Different Cell Lines

The intrinsic cytotoxicity of RGD‐IONs in U87, MDA‐MB‐231 and bEnd.3 cell lines were evaluated with CCK‐8 assays. 5 × 10^3^ cells were seeded into 96‐well plates and cultured for 24 h, and followed by incubation with different concentrations of Fe (Fe = 200, 100, 50, 25, 12.5, 6.25, and 3.12 µg mL^−1^) of sterilized RGD‐IONs at 37 °C for 1, 2, and 3 d, respectively. The cell viability was measured by using CCK‐8 assay kit under the microplate reader with absorbance at 450 nm. The intrinsic cytotoxicity of 22 nm RGD‐MNPs with different iron concentrations on U87 cells was also detected with the CCK‐8 assays. The final concentrations were 1600, 800, 400, 200, 100, 50, and 25 µg mL^−1^ respectively.

##### Localization of RGD‐IONs in Cells

U87 cells were seeded on Φ 60 mm dishes with a density of 2 × 10^5^ cells per dish and cultured for 24 h, followed by the addition of RGD‐IONs with 50 µg Fe in each culture dish. After coincubation for 24 h, cells were collected by centrifugation. Afterwards, cells were fixed with 2.5% glutaraldehyde and 1% aqueous osmium tetroxide orderly, and then cells were dehydrated by acetone and embedded in Epon Araldite resin. After that, ultrathin sections (100 nm) were selected from the cell aggregates carefully, stained with 4% uranyl acetate and 0.2% Reynolds lead citrate orderly, and then the samples were air‐dried. Finally, bio‐TEM images were captured by FEI Tecnai F30 microscope at 300 kV.

##### Quantification of RGD‐IONs Internalized in Cells

The quantification of Fe uptake by U87 cells was measured by ICP‐OES. Briefly, 1 × 10^5^ cells were seeded on 12‐well plate and incubated with RGD‐IONs with Fe 25 and 50 µg mL^−1^ respectively for 2, 4, 6, 8, 12, 24 h. Then, cells were digested and collected. After dissolved in aqua regia medium, the content of Fe was quantified by ICP‐OES. The internalization amount of 22 nm RGD‐MNPs (25 µg mL^−1^) and ferumoxytol (200 µg mL^−1^) were also detected following the same procedure.

##### Therapeutic Effect of MTIT Treatment In Vitro

U87, MDA‐MB‐231, bEnd.3 cells, astrocytes, and 3T3 cells were planked on Φ35 mm confocal dish with a density of 5 × 10^4^ and cultured 24 h for attachment. Four groups were set for each cell line including materials control (MC), MF, MH and MTIT. RGD‐IONs with Fe 25 µg were added to each confocal dish and cultured for another 24 h for uptake. Then MF, MH, and MTIT treatment were carried on each group. The MF treatment was using RMF with 40 mT, 15 Hz, and 15 min. The MH treatment was using AMF with 150 Oe, 375 kHz, and 6 min. The MTIT group was performed with MF 6 min and MH 15 min orderly. The cell viability was calculated by using CCK‐8 assay kit. Each group has five duplicates. Thermal imaging of cells during MH and MTIT were collected by a NIR thermal camera every 2 min.

In addition, live/dead cells staining was performed to qualitatively evaluate the therapeutic effect of MTIT (Figure S8, Supporting Information). Four groups were set including MC, MF, MH and MTIT. 5 × 10^4^ U87 cells were seeded on the Φ35 mm confocal dish for 24 h. Then, RGD‐IONs with 25 µg Fe were added to each group. After coincubation for another 24 h, cells were treated with different treatment. Calcein‐AM/PI staining was used to evaluate the therapeutic effect posttreatment 24 h. The concentration of calcein‐AM and PI were 2 × 10^−6^ and 1.5 × 10^−6^ m respectively, staining for 30 min. Finally, the images were captured by the Live Cell Imaging System (EVOS, Life Technologies).

Moreover, annexin V‐PI staining was used to differentiate the mode of cell death. The cells were seeded with 5 × 10^4^ U87 cells on Φ35 mm confocal dish and cultured for 24 h. After addition of RGD‐IONs with 25 µg Fe to each group, different treatment was carried on. After 12 h, cells were stained by annexin V‐FITC/PI sequentially to differentiate the live, necrotic, late apoptotic, and early apoptotic cells. The concentration of annexin V‐FITC and PI were diluted to appropriate concentrations, and dyeing time were both 15 min. At last, the fluorescence intensities of annexin V‐FITC and PI were quantitatively detected by flow cytometry (Attune NxT acoustic focusing Cytometer).

##### Synergistic Effect of MTIT

To verify the synergistic effect of MTIT, two sets of experiments were carried out. 5 × 10^4^ U87 cells were seeded on the Φ35 mm confocal dish for 24 h followed by the addition of RGD‐IONs with 25 µg Fe for each group for another 24 h incubation. Then MF treatments with different time (0, 5, 10, 15, 20, 25, 30 min) were performed. Meanwhile, MTIT groups were performed with different MF time (0, 5, 10, 15, 20, 25, 30 min) and the fixed MH time of 6 min. The frequency of RMF and AMF were 15 Hz and 375 kHz respectively. Posttreatment 24 h, the cell viabilities were evaluated by CCK‐8 assay kit. Similar experimental designs were applied in which MH groups with different time (0, 2, 4, 6, 8, 10 min) and MTIT groups with fixed MF time (15 min) and different MH time (0, 2, 4, 6, 8, 10 min), calculating by CCK‐8 assay kit. The synergistic effect index Q was calculated according to the following formula

(1) $$\begin{array}{ccc} Q & = & \frac{E_{\mathrm{MTIT}}}{E_{\mathrm{MF}}+\left(1-E_{\mathrm{MF}}\right)E_{\mathrm{MH}}}\;\begin{array}{c} E_{\mathrm{MF}}:\;\text{therapeutic}\;\text{effect}\;\text{of}\;\text{MF}\\ E_{\mathrm{MH}}:\;\text{therapeutic}\;\text{effect}\;\text{of}\;\text{MH}\\ E_{\mathrm{MTIT}}:\;\text{therapeutic}\;\text{effect}\;\text{of}\;\text{MTIT} \end{array}\\ Q & \geq & \text{1.15:}\;\text{synergistic}\;\text{effect} \end{array}$$

##### The Therapeutic Effect of MTIT of Commercial Ferumoxytol

The size and zeta potential were characterized by DLS. The intrinsic toxicity of ferumoxytol with different concentration of Fe (0, 25, 50, 100, 200, 400, 800,1600 µg mL^−1^) was quantified by CCK‐8 assay kit after incubation with U87 cells for 24 h. The magnetic heating property was also carried out with different concentration of Fe of ferumoxytol recorded by NIR thermal imaging. The therapeutic effect was used 5 × 10^4^ U87 cells and ferumoxytol with 200 µg Fe, followed by the treatment with same parameters in RGD‐IONs treatment.

##### The Mechanism Study on Changes of Cell Structure

To investigate the mechanism of synergistic effect, bio‐TEM and live cell imaging system were first applied to observe the morphology and inner structure changed. U87 cells were seeded on Φ 60 mm dishes with a density of 2 × 10^5^ cells per dish and cultured for 24 h, followed by the addition of RGD‐IONs with 50 µg Fe in each culture dish for another 24 h. Cells were treated with different groups, MH with frequency of 375 kHz and time of 6 min, MF with frequency of 15 Hz and time of 15 min. Afterwards, cells were collected and underwent the sample preparation and observation of bio‐TEM. Besides, live cell imaging system was used to observed the morphology change at different time post treatment.

##### Detection of Lysosomal Membrane Permeabilization

Based on the localization of RGD‐IONs in cells, lysosomal membrane permeabilization was explored. According to the previous work by Aits et al., the plasmid EGFP‐Gal3 (pCDH‐EF1‐EGFP‐LGALS3) created by cloning human LGALS3 (isoform 1) sequences with lentiviral pCDH‐EF1‐MCS‐IRES‐Neo vector (Systems Biosciences, CD533A‐2) containing N‐terminal EGFP tags. U87 cells were planked on Φ 35 mm confocal dishes with a density of 1 × 10^5^ cells per dish and cultured for 24 h, followed by transfected by above plasmid using lipofectamine 6000 for another 24 h for the expression of LGALS3‐GFP. Afterwards, RGD‐IONs with Fe 25 µg were added in each confocal dish and coincultured 24 h for endocytosis. Then, different treatments were performed with the parameters shown before. The number and intensity of highspots occurred after treatment were observed via an inverted fluorescence microscope within live cell imaging system (EVOS, Life Technologies), reflecting the lysosomal membrane permeabilization. Besides, lysotracker was also used to detect the integrity of lysosomal membrane, whose intensity depends on the acidic environment of lysosome. The cell preparation, RGD‐IONs addition and treatment were performed. After treatment, lyso‐tracker red with final concentration of 50 × 10^−9^ m was used to stain cells for 60 min and observed by live cell imaging system (EVOS, Life Technologies).

##### Characterization of Mitochondrial Membrane Potential

The preparation of cells, addition of RGD‐IONs and treatment of cells were consistent with the MTIT experiment. After treatment 4 h, JC‐1 with final concentration of 2 × 10^−6^ m was used to stain the cells for 30 min to detect the change of mitochondrial membrane potentials. The green and red fluorescence representing J‐monomer and J‐aggregates were observed and captured under the live cell imaging system (EVOS, Life Technologies).

##### Characterization of Cytoskeleton Structure

The integrity of F‐actin was characterized by Phalloidin‐iFluor 555 conjugate. The preparation of cells, addition of RGD‐IONs and treatment of cells were performed orderly. Just after treatment and after treatment 24 h, cells were fixed with 4% paraformaldehyde for 10 min, followed by Phalloidin‐iFluor 555 conjugate staining for 90 min and Hoechst 33 342 staining for 15 min, labeling F‐actin and nucleus respectively (Figure S18, Supporting Information). The cells were captured by live cell imaging system (EVOS, Life Technologies).

##### Reuptake of RGD‐IONs Released from the Damaged Cells

RGD‐IONs with iron concentration of 25 µg mL^−1^ were first internalized by MDA‐MB‐231 without RFP expression, subsequently, different treatments were performed to induce cell death. The parameters of RMF and AMF were same as before. Then, the cell fragments and leaked RGD‐IONs were collected and coincubated with the fresh RFP labeled MDA‐MB‐231 cells. After coincubation of 24 h, the cells were stained by Prussian blue dyes. The images were captured to distinguish the RGD‐IONs inside or outside of the cells (the scale bar: 200 µm). After reuptake of RGD‐IONs, different treatments were performed. The parameters of RMF and AMF were same as before. The cell viability was measured via CCK‐8 kit.

##### DHE Staining

To classify the ROS, superoxide anion probe dihydroethidium (DHE) was applied. Cell seeding and RGD‐IONs adding were followed by the DHE staining with final concentration of 1 × 10^−6^ m for 30 min, and then different treatments were performed. The red fluorescence of cells was observed in live cell imaging system (EVOS, Life Technologies)

##### Programmed ROS Generation

The overall level of ROS was quantified by using DCFH‐DA via flow cytometry. The cell preparation and RGD‐IONs addition were same as that in MTIT treatment. Then, cells were stained with 10 ×10^−6^ m DCFH‐DA for 30 min. After washing three times, different treatments containing MF groups (0, 3, 6, 9, 12, 15 min) and MTIT groups with varied MF time (0, 3, 6, 9, 12, 15 min) and fixed MH time (6 min) were carried out. Then, cells were digested and collected for ROS detection by flow cytometry. At the meanwhile, cell viability was detected by using CCK‐8 assay kit after treatment 24 h, without DCFH‐DA staining.

##### ROS Inhibition for Enhanced Cell Viability

NAC, a scavenger of ROS, was used to eliminate ROS generated during MTIT. Cell preparation and RGD‐IONs addition were same as before, followed with or without 2.5 × 10^−3^ m NAC pretreatment 2 h. Then MTIT treatments with varied MF time (0, 3, 6, 9, 12, 15 min) and fixed MH (6 min) were executed. Afterwards, cells were collected to detect intracellular ROS level by flow cytometry. In addition, NAC was used to reverse the killing effect of ROS. There was a pretreatment of 2.5 × 10^−3^ m NAC for 2 h, followed by MF, MH, or MTIT treatment. After treatment 24 h, cell viability was evaluated by CCK‐8 assay kit.

##### In Vivo Experiments on U87 Glioblatoma Model

All animal experiments were performed in accordance with the guidelines of the animal care and use committee at Tongji University. Glioblastoma U87 tumor‐burdened nude mice model were established by the subcutaneous injection of 5 × 10^6^ U87 cells on the right flank of nude male mice. When the tumor size grew to 100 mm^3^, the mice were divided into four groups (five mice per group) randomly containing MC, MF, MH, MTIT. Afterwards, 5 mg kg^−1^ RGD‐IONs with 25 µL were intratumoral injected into tumors at day 0, 2, and 4. The treatments were applied at day 1, 3, 5, 6, 7, 8, and 9 for seven times in total, accompanied by the temperature recorded. The parameters of RMF for MF treatment were frequency of 15 Hz, strength of 40 mT and duration of 30 min for each time, while for MH treatment, the parameters of AMF were frequency of 375 kHz, strength of 150 Oe and duration of 6 min for each time. MTIT was combined MF and MH treatment orderly. The body weight and tumor volume were measured daily for 3 weeks. The tumor volume was calculated following the equation: *V* = length × width^2^ / 2. The pictures of mice were captured at day 5 and 21. Then, the mice were sacrificed, and the tumors were collected to record weight, capture pictures and subjected to histological analysis using H&E staining, Prussian blue staining. The organs were also collected to do histological analysis.

##### In Vivo Experiments on MDA‐MB‐231 Breast Cancer Model

A triple negative breast cancer MDA‐MB‐231‐bearing subcutaneous mice model was established by injection of 1 × 10^6^ MDA‐MB‐231 cells into the right flank of male nude mice. When the tumor volume reached to 100 mm^3^, the mice were randomly divided into four groups (five mice per group): PBS, MF, MH and MTIT. Then, 25 µL 5 mg kg^−1^ RGD‐IONs were intratumorally injected into tumors at day 0 and 2. The treatments were performed at day 1, 3, 4, 5 for four times and recorded temperature. The parameters of RMF and AMF were consistent with those in U87 model. The body weight and tumor size were daily recorded for 3 weeks. Then, the mice were sacrificed and the organs and tumor tissues were collected and subjected to HE staining and Prussian blue kits.

##### Statistical Analysis

All data statistical analyses were performed to use the software of GraphPad Prism 5. The data number for each group was ≥3 and numerical data were reported as Mean ± SD. *p* value was considered as statistically significant at *p < 0.05, **p < 0.01, ***p < 0.001.

## Conflict of Interest

The authors declare no conflict of interest.

## Supporting information

Supporting Information

Supporting Information
