# Supplementary material for: Programmable ROS‐Mediated Cancer Therapy via Magneto‐Inductions
Source: Adv Sci (Weinh). 2020 May 9;7(12):1902933. doi: 10.1002/advs.201902933 (PMC7312334; doi:10.1002/advs.201902933)
Supplement: Supplementary file 1 — Supporting Information [file ADVS-7-1902933-s002.pdf]

Supporting Information

Programmable ROS-Mediated Cancer Therapy via Magneto-Inductions

*Jiaojiao Wu, Peng Ning, Rui Gao, Qishuai Feng, Yajing Shen, Yifan Zhang, Yingze Li, Chang Xu, Yao Qin, Gustavo R. Plaza, Qianwen Bai, Xing Fan, Zhenguang Li, Yu Han, Maciej S. Lesniak, Haiming Fan, and Yu Cheng***

J. Wu, Prof. Y. Cheng
Institute for Regenerative Medicine, Institute for Translational Nanomedicine, Shanghai East Hospital, Tongji University School of Medicine, 1800 Yuntai Road, Shanghai, 200123, China
Collaborative Innovation Center for Brain Science, Tongji University, Shanghai 200092, China
Email: yucheng@tongji.edu.cn

P. Ning, R. Gao, Q. Feng, Y. Shen, Y. Li, C. Xu, Dr. Y. Qin, Q. Bai, X. Fan, Z. Li
Institute for Regenerative Medicine, Institute for Translational Nanomedicine, Shanghai East Hospital, Tongji University School of Medicine, 1800 Yuntai Road, Shanghai, 200123, China

Y. Zhang, Prof. H. Fan
College of Chemistry and Materials Science, Northwest University, Xi'an 710127, China

Prof. G. R. Plaza
Center for Biomedical Technology, Universidad Politécnica de Madrid, Pozuelo de Alarcón 28223, Spain

Y. Han, Dr. M. S. Lesniak
Feinberg School of Medicine, Northwestern University, 676 North Saint Clair Street, Suite 2210, Chicago, Illinois 60611, United States

Figure:

S1. Synthetic process and characterization of MNPs.

S2. Theoretical calculation of mechanical force generated under RMF.

S3. Magnetic heating characterizations of RGD-IONs.

S4 Intrinsic cytotoxicity of RGD-IONs on U87, MDA-MB-231 and bEnd.3 cells.

S5. Colocalization of RGD-IONs and lysosomes.

S6. Qualitative analysis of therapeutic efficacy *in vitro*.

S7. Characterizations of 22 nm cubic MNPs' physical properties.

S8. Intrinsic and treated cytotoxicity of 22 nm RGD-MNPs.

S9. Compare of the internalization of nanomaterials in U87 cells.

S10 Characterizations of commercial Ferumoxytol and the therapeutic effect of Ferumoxytol coupled with magnetic fields.

S11. Intrinsic and treatment cytotoxicity of RGD-IONs on Astrocytes and 3T3 cells.

S12. Temperature recorded during MTIT and MH groups in *in vitro* experiments.

S13. Cell viability of U87 cells in different treated groups.

S14. Cell structure evaluation.

S15. The alignment of RGD-IONs in MTIT group.

S16. Subcellular structure changed characterization.

S17. Colocalization of RGD-IONs and mitochondria based on Bio-TEM imaging.

S18. Cytoskeleton change of U87 cells after different treatment.

S19. Biochemical signals detection, involving ROS and HSP70.

S20. Animal experiments in U87-bearing mouse model.

S21. Animal experiments in triple negative breast cancer model.

S22. Reuptake of RGD-IONs in MDA-MB-231@RFP cells.

S23. Compare of the cell viability of MTIT on MDA-MB-231 cells mediated with natural internalized and reinternalized RGD-IONs.

Table:

S1. Synergistic effect of MTIT with varied MF time.

S2. Synergistic effect of MTIT with varied MH time.

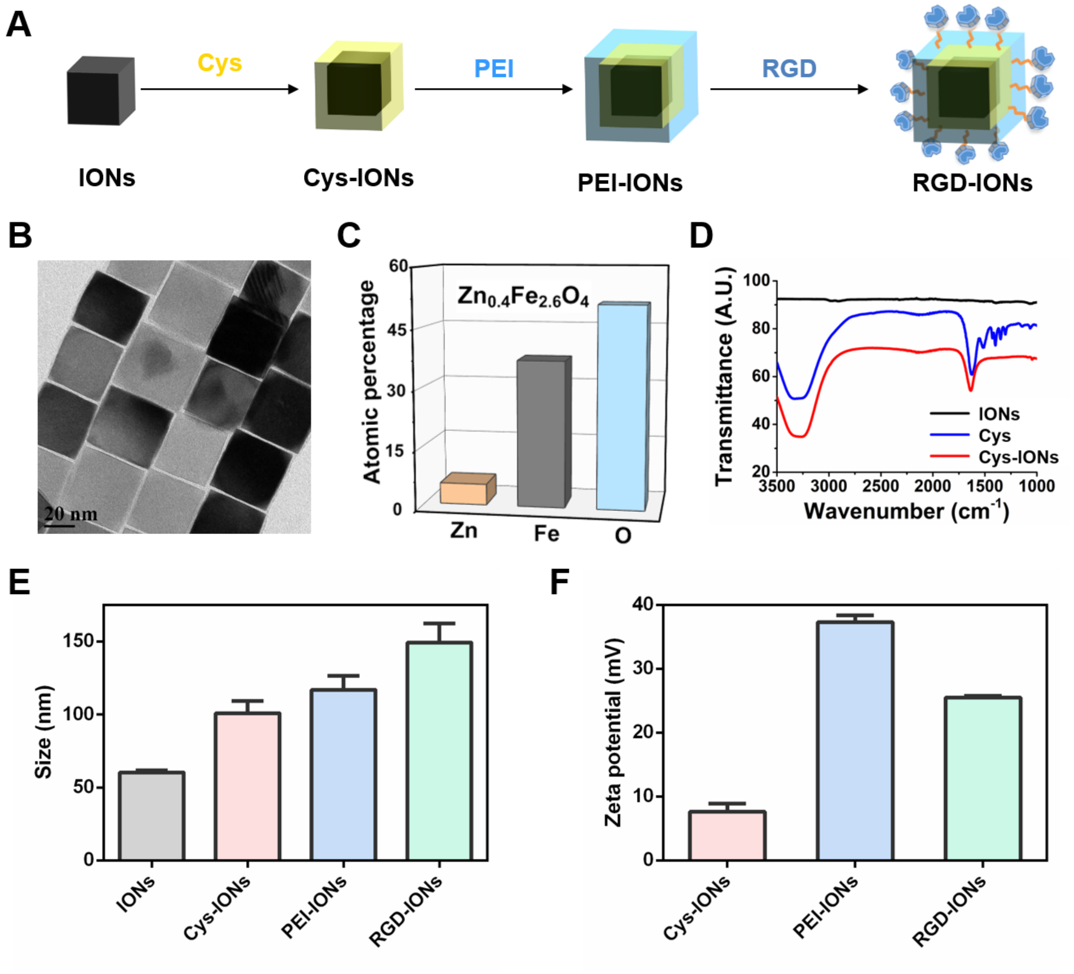

Figure S1. Synthetic process and characterization of MNPs. (A) Schematic illustration of synthetic process. **(B)** TEM image of IONs. **(C)** EDS analysis of IONs demonstrating their composition. **(D)** FTIR spectrum of Cys-IONs, confirming the successful modification of Cysteine on IONs' surface. **(E)** DLS analysis of average size of MNPs with different modifications. **(F)** Zeta potential of intermediate and final MNPs.

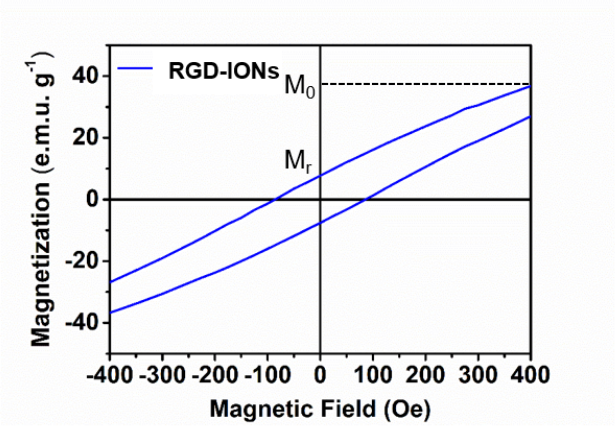

Figure S2. Theoretical calculation of mechanical force generated under 40 mT RMF. It was calculated using the following equation .

$$\tau = N \cdot mass_{single} \cdot (M_r + (M_0 - M_r) \cdot \cos\theta) \cdot B_0 \cdot Sin\theta$$

where $mass_{single}$ is the mass of one IONs, M_r and M_0 are respectively the retentiveness and the magnetization for the applied magnetic field B_0 = 40 mT, and θ is the angle between the magnetic field and the longitudinal direction of the aggregate. From the magnetization curve of RGD-IONs above, under the magnetic field of 40 mT, M_0 = 36.4 emu·g^{-1} and M_r= 7.3 emu/g. A maximum value of the torque could be obtained for $\theta \approx 50°$ and equal to

$$\tau_{max} = 0.55 \cdot N \cdot mass_{single} \cdot M_0 \cdot B_0$$

$$\tau_{max} = N * 0.897\, pN \cdot \mu m$$

$$F_{max} = \tau_{max} / \frac{2L}{3}$$

For N=5, the magnitude of the force induced by the elongated nanocubes was estimated to be 22.5 pN.

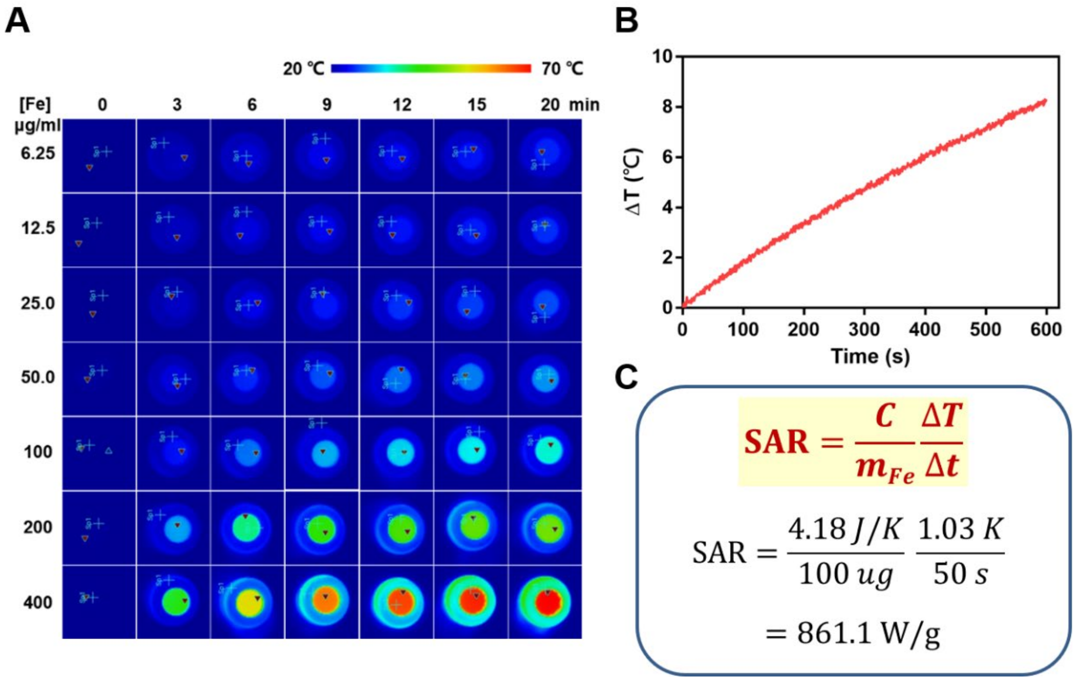

Figure S3. Magnetic heating characterizations of RGD-IONs. (A) Infrared thermal images of RGD-IONs dispersed in water with different iron concentrations under AMF treatment with frequency of 375 kHz for 20 min. (**B)** Temperature raised curves of RGD-IONs with iron concentration of 100 μg/mL dispersed in 1% agarose gel under AMF with frequency of 399 kHz (330 Oe) for 10 min. The temperature was recorded by optical fiber. (**C)** Calculation of SAR value of RGD-IONs.

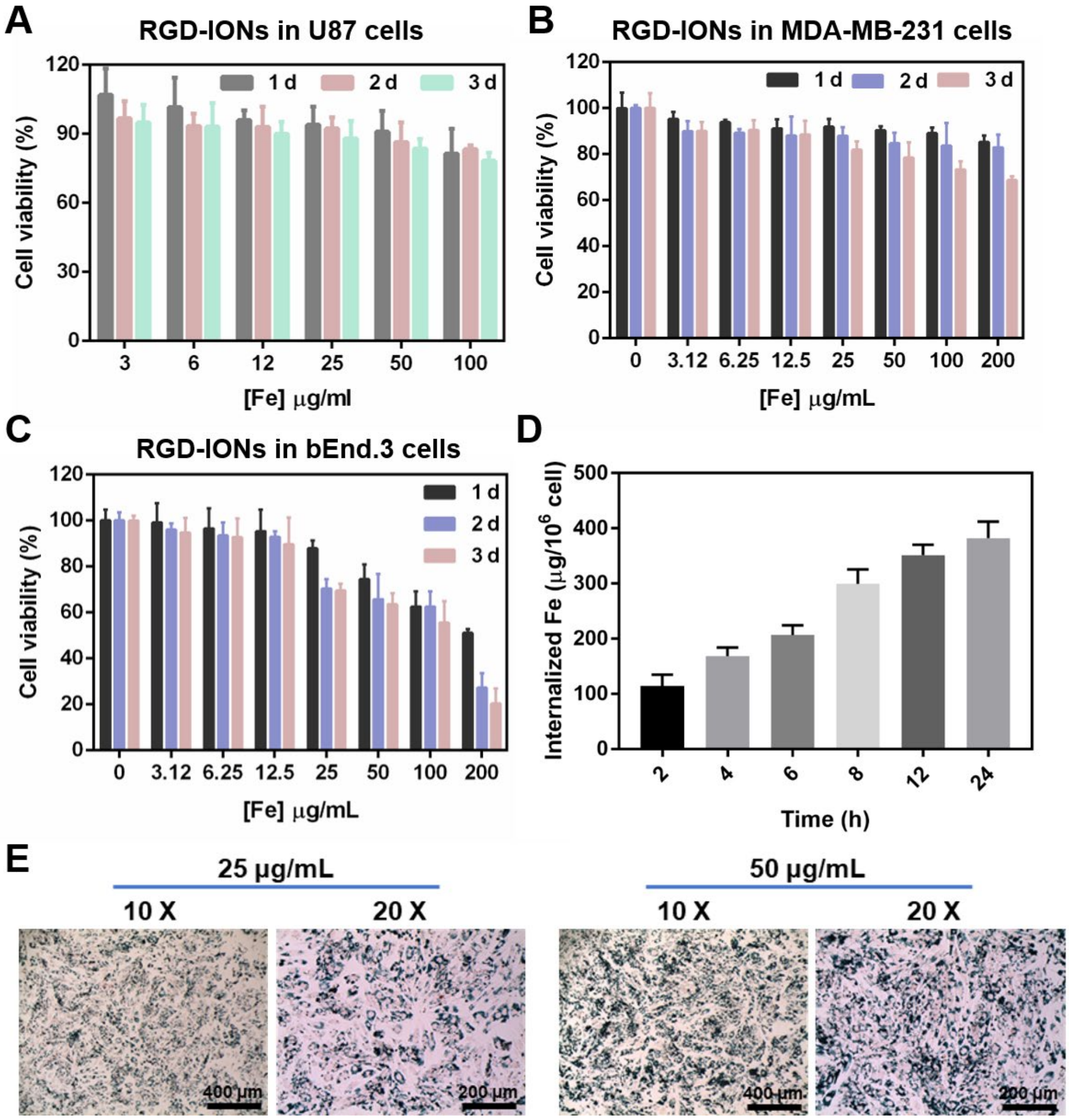

Figure S4. Intrinsic cytotoxicity of RGD-IONs on U87, MDA-MB-231 and bEnd.3 cells. (A, B and **C)** The cell viability of U87 cells, MDA-MB-231 cells and bEnd.3 cells treated with RGD-IONs with different iron concentrations for 1 d, 2 d and 3d. The cell viability was measured by CCK-8 kit. (**D)** Intracellular iron content of U87 with different incubation periods of RGD-IONs. The RGD-IONs were co-cultured with U87 cells for 2, 4, 6, 8, 12 and 24 h. And the iron content was analyzed via ICP-MS. **(E)** Prussian blue staining of bEnd.3 cells after coincubation with RGD-IONs with different iron concentrations for 24 h.

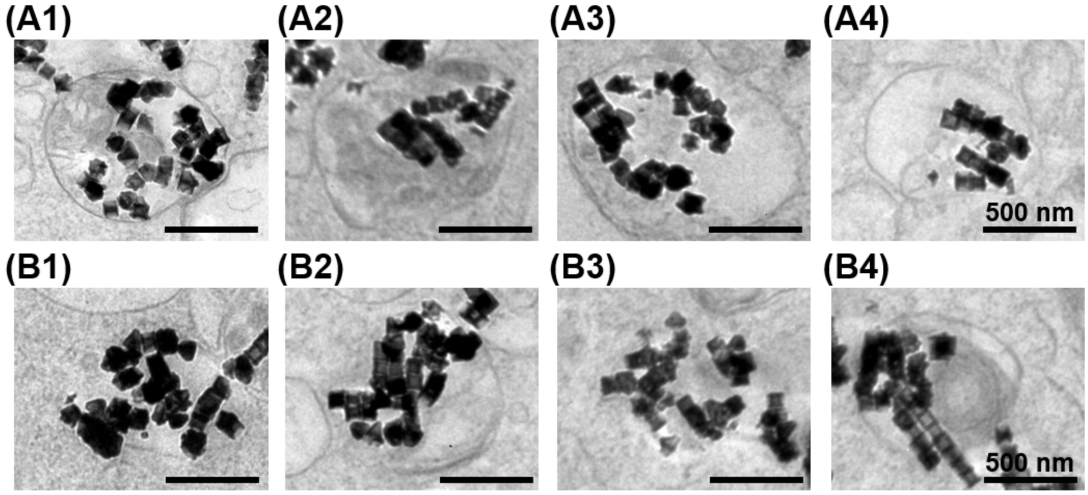

Figure S5. Colocalization of RGD-IONs and lysosomes. The Bio-TEM images were captured for U87 cells incubated with 25 μg/mL RGD-IONs for 24 h (scale bar: 500 nm). **(A1-A4)** RGD-IONs were located in lysosomes. **(B1-B4)** RGD-IONs could escape from lysosomes to cytoplasm.

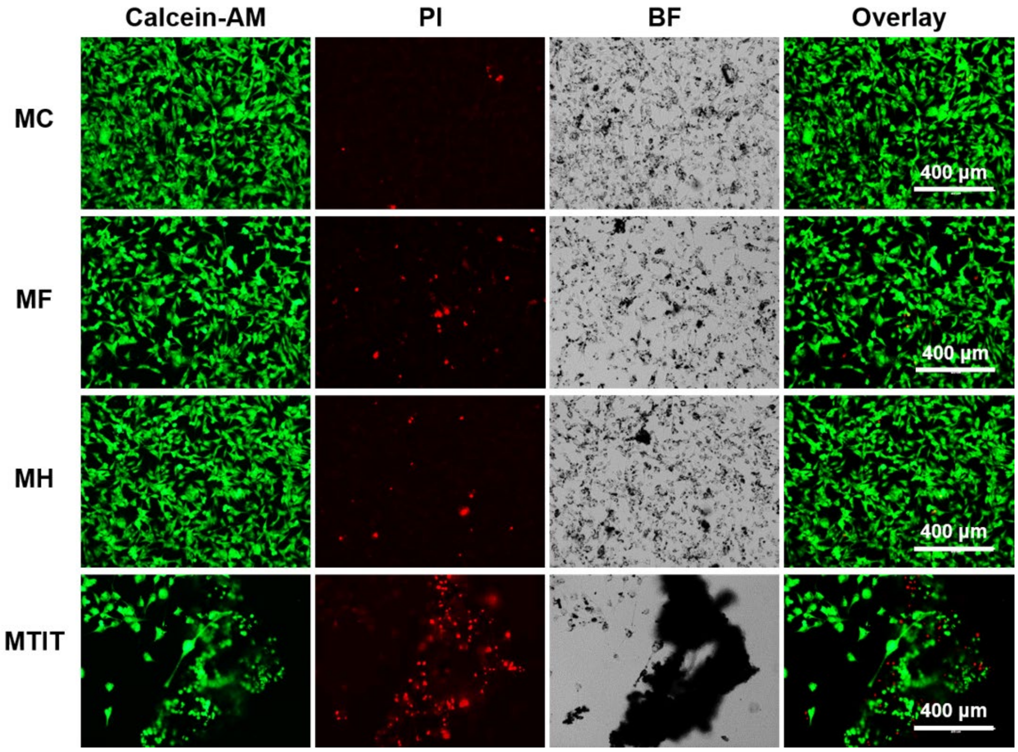

Figure S6. Qualitative analysis of therapeutic efficacy *in vitro*. The live/dead staining of U87 cells after different treatment was evaluated by calcein-AM/PI kit.

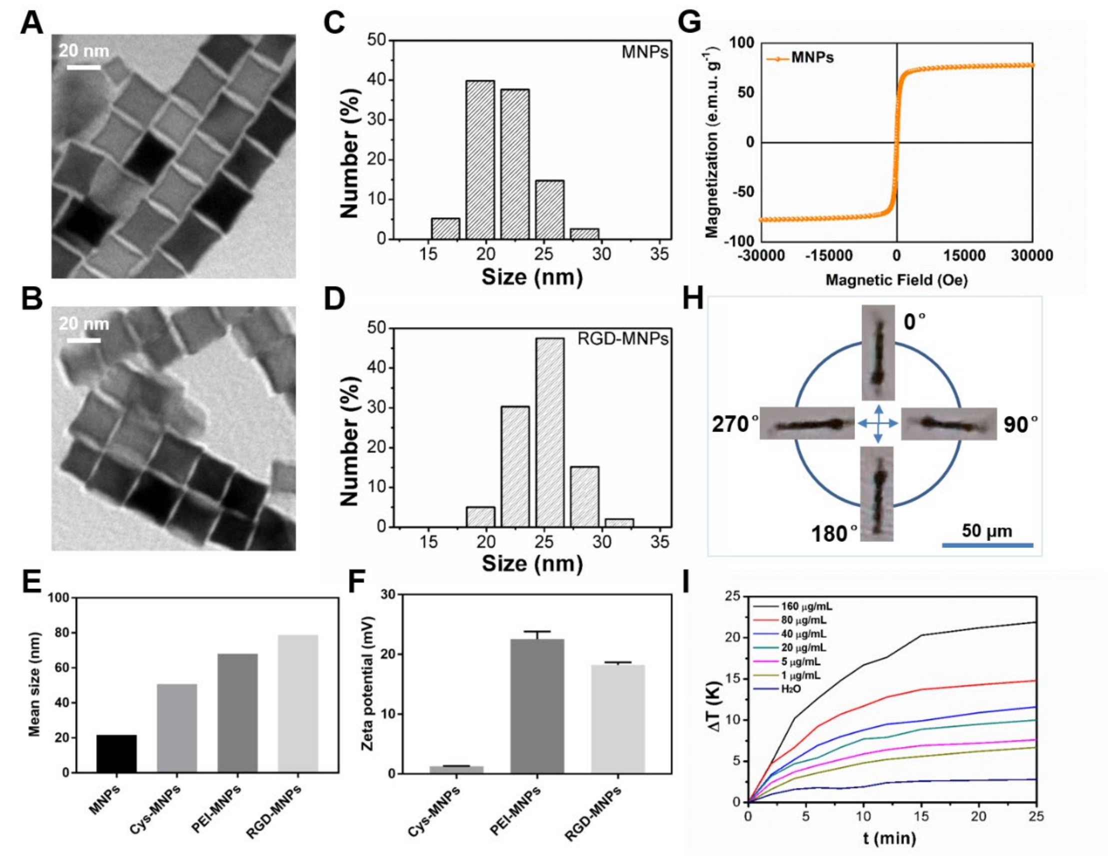

Figure S7. Characterizations of 22 nm cubic MNPs' physical properties. (A) TEM image of MNPs synthesized (scale bar: 20 nm). **(B)** TEM image of RGD-MNPs (scale bar: 20 nm). **(C)** The size distribution of MNPs measured by Nano Measurer software. **(D)** The size distribution of RGD-MNPs measured by Nano Measurer software. **(E)** The DLS analysis of average size of 22 nm cubic MNPs with different modifications. **(F)** The zeta potential of intermediate and final MNPs. **(G)** Magnetic properties represent by M-H curves of MNPs (Temperature: 298 K). **(H)** Optical images showing the assembly of RGD-MNPs and rotation under RMF with frequency of 0.1 Hz. **(I)** Magnetic thermal properties of RGD-MNPs with different concentrations under AMF with frequency of 375 kHz for 25 minutes.

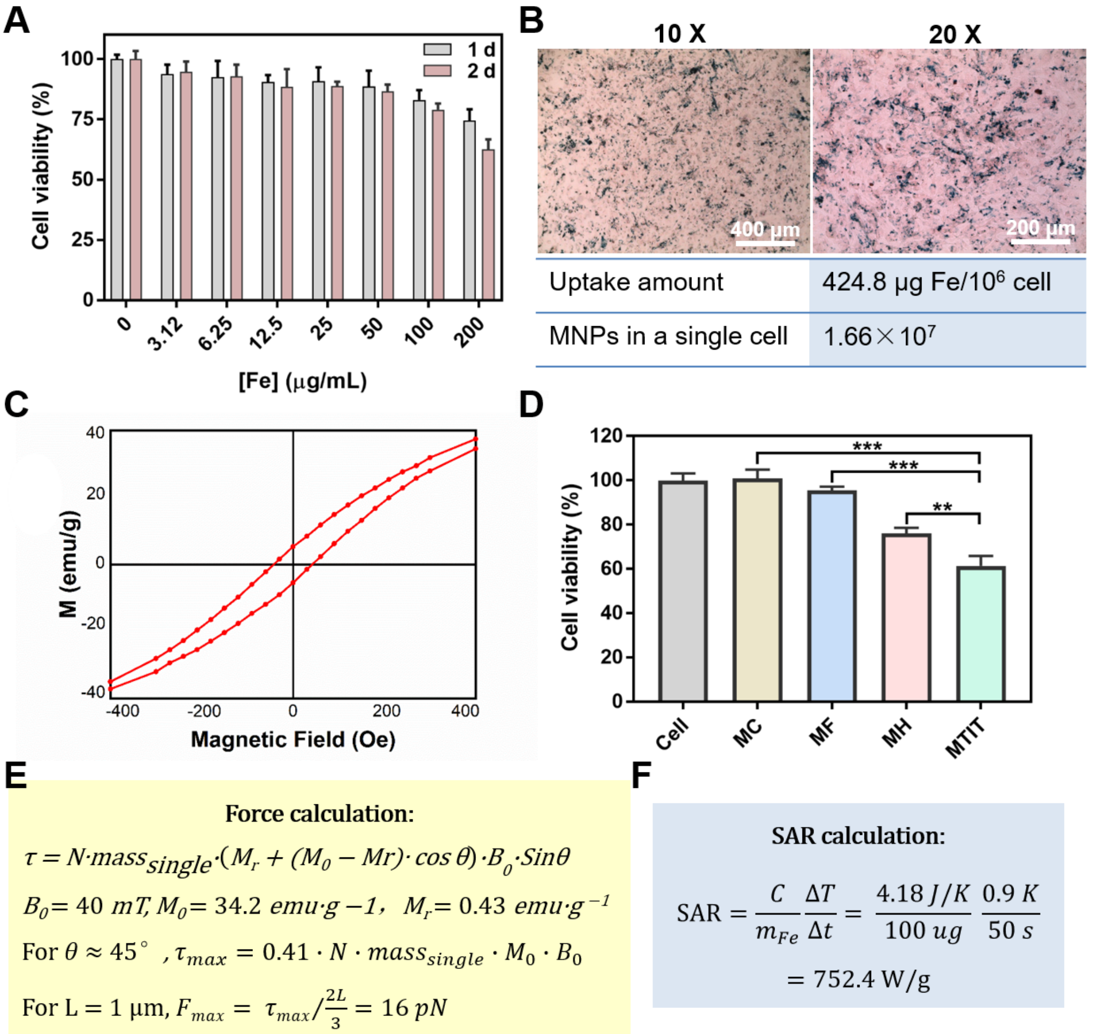

E

Force calculation:

$\tau = N \cdot mass_{single} \cdot (M_r + (M_0 - Mr) \cdot \cos\theta) \cdot B_0 \cdot Sin\theta$

$B_0 = 40\ mT, M_0 = 34.2\ emu \cdot g - 1, \quad M_r = 0.43\ emu \cdot g^{-1}$

For $\theta \approx 45^\circ$, $\tau_{max} = 0.41 \cdot N \cdot mass_{single} \cdot M_0 \cdot B_0$

For L = 1 μm, $F_{max} = \tau_{max} / \frac{2L}{3} = 16\ pN$

F

SAR calculation:

$$\text{SAR} = \frac{C}{m_{Fe}} \frac{\Delta T}{\Delta t} = \frac{4.18\ J/K}{100\ ug} \frac{0.9\ K}{50\ s}$$

$$= 752.4\ \text{W/g}$$

Figure S8. Intrinsic and treated cytotoxicity of 22 nm RGD-MNPs. (A) The cell viability of U87 cells treated with RGD-MNPs with different iron concentrations for 1 d and 2 d. **(B)** Optical images of RGD-MNPs with iron concentrations of 25 and 50 μg/mL internalized in U87 cells after co-incubation of 24 h. **(C)** Zoomed M-H curve showing the magnetic property of MNPs measured by VSM at temperature of 298 K. **(D)** The cell viability of U87 cells after different treatments. RGD-MNPs with iron concentration 25 μg/mL were co-incubated with U87 for 24 h followed by different treatments. The parameters of MF and were 40 mT and 15 min, and 375 kHz, 150 Oe and 6 min for MH treatment. **(E)** Theoretical calculation of the mechanical force generated under 40 mT RMF. **(F)** Calculation of SAR values according to the temperature raised curves and the equation. The AMF parameters were 375 kHz and 12 kA/m.

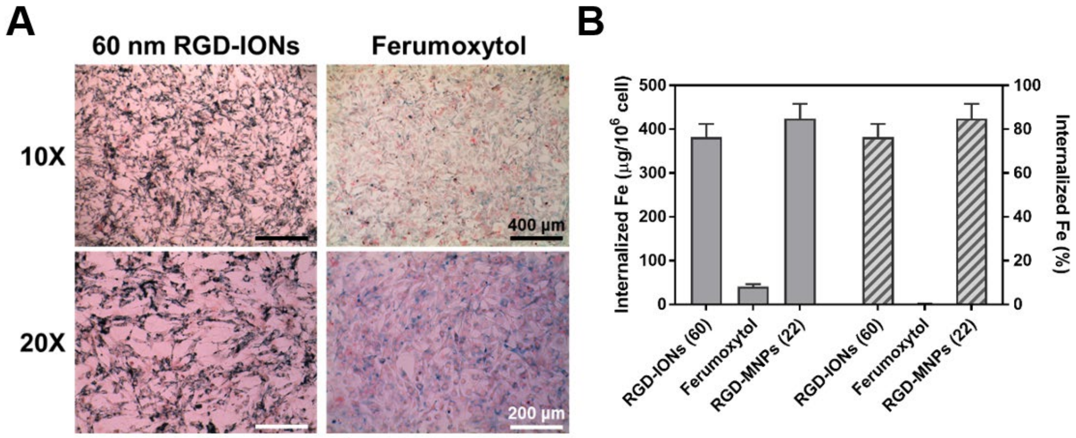

Figure S9. Compare of the internalization of nanomaterials in U87 cells. (A) Prussian blue staining of U87 cells after coincubation with 60 nm RGD-IONs (25 μg/mL) and Ferumoxytol (200 μg/mL) for 24 h (the black scale bar: 400 μm and the white scale bar: 200 μm). **(B)** Quantitative analysis of 60 nm RGD-IONs, Ferumoxytol and 22 nm RGD-MNPs internalized by U87 cells after coincubation of 24 h. The original Fe concentrations were 25 μg/mL, 200 μg/mL and 25 μg/mL respectively. The internalized Fe mass and internalized Fe percentage were shown in left y axis and right y axis, respectively.

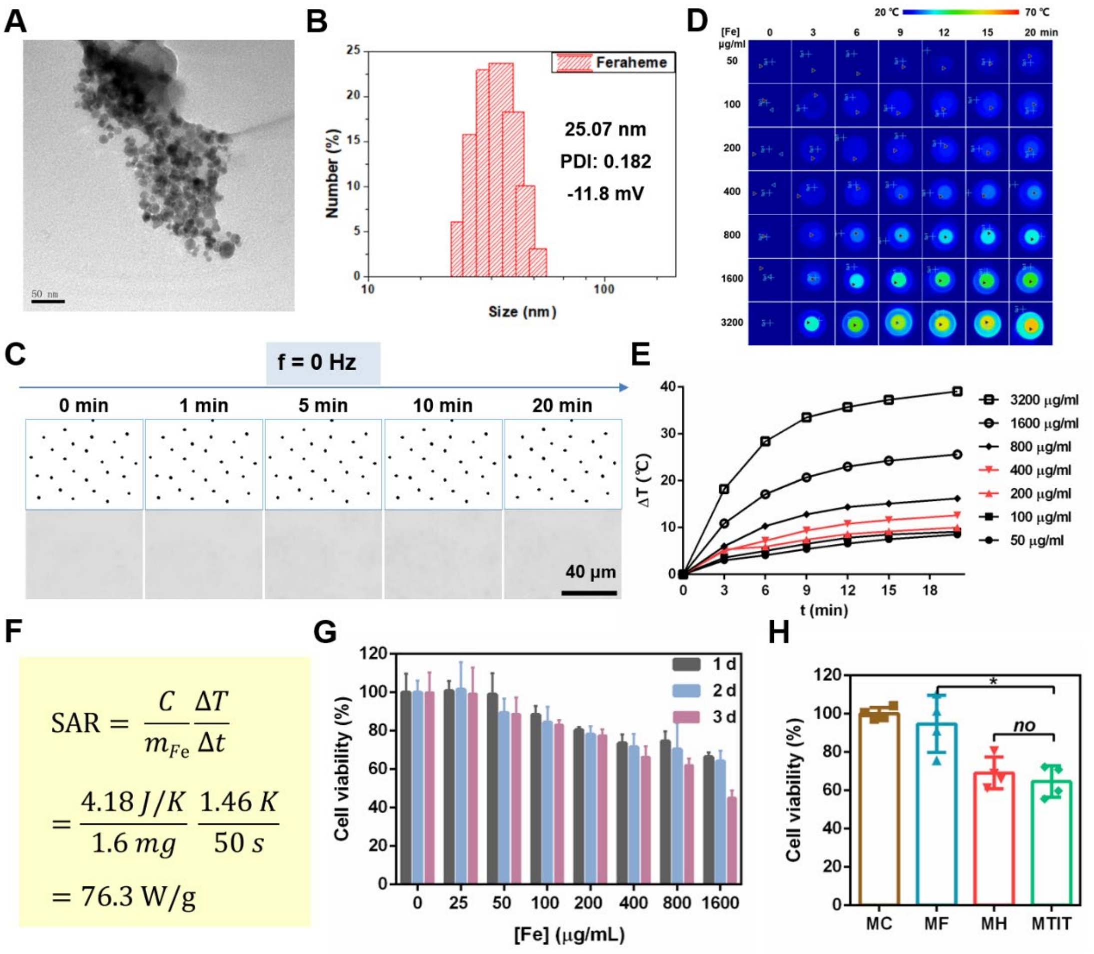

Figure S10. Characterizations of commercial Ferumoxytol and the therapeutic effect of Ferumoxytol coupled with magnetic fields. (**A)** TEM image. (**B)** DLS analysis of average size, PDI and zeta potential. **(C)** The optical images showing the assembled ability of Ferumoxytol under RMF with strength of 40 mT and frequency of 0 Hz (the scare bar: 40 μm). **(D)** IR thermal images of Ferumoxytol with different concentrations. (**E)** Temperature raised curves of Ferumoxytol with different concentrations. (**F)** The calculation of SAR value. **(G)** The intrinsic toxicity of Ferumoxytol on U87 cells. The cell viability was measured by CCK-8 kit after conincubation with Ferumoxytol with different iron concentrations for 1 d, 2 d and 3d. **(H)** The therapeutic effect of MTIT mediated by Ferumoxytol on U87 cells. The parameters of RMF and AMF were 15 Hz,15 min and 375 kHz, 6 min, respectively.

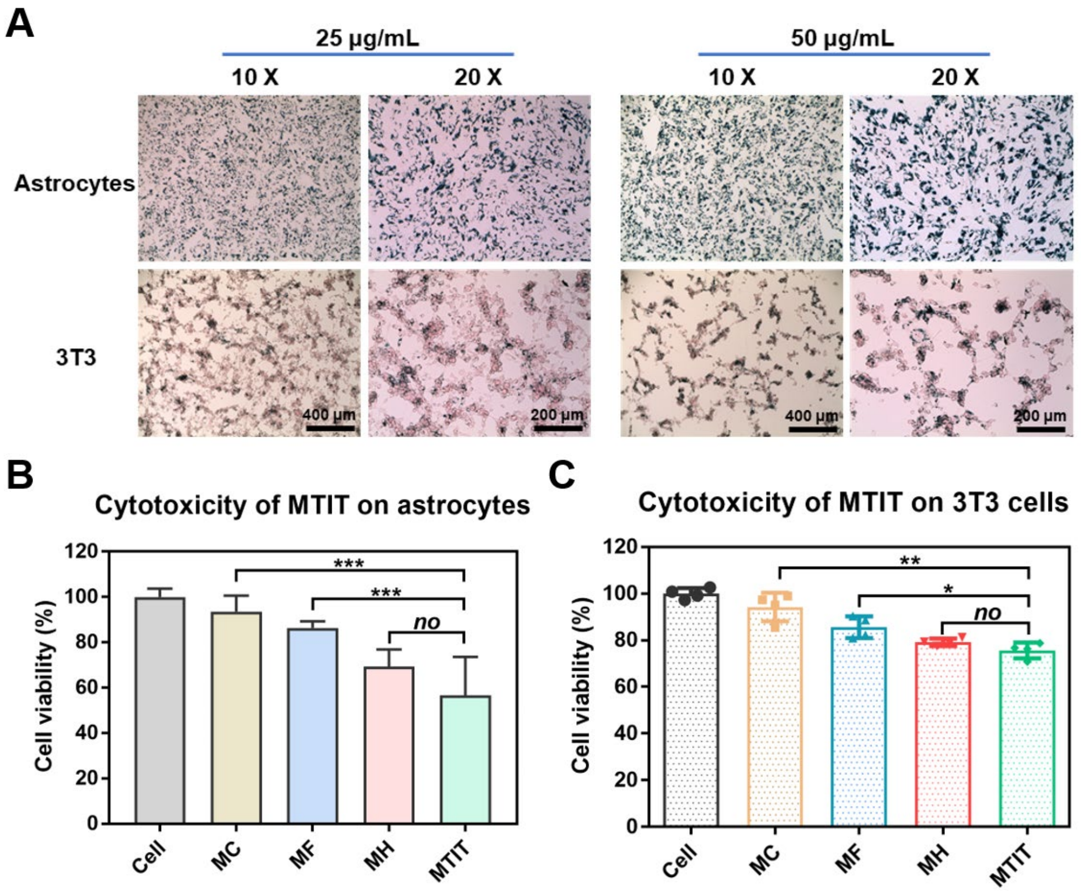

Figure S11. Intrinsic and treatment cytotoxicity of RGD-IONs on astrocytes and 3T3 cells. (A) Prussian blue staining of astrocytes and 3T3 cells after coincubation with RGD-IONs for 24 h. The concentrations of RGD-IONs were 25 and 50 μg/mL Fe, respectively. The scale bars were 400 and 200 μm, respectively. **(B** and **C)** The cell viability of astrocytes and 3T3 cells after different treatments measured by CCK-8 kit. The Fe concentration was 25 μg/mL. Statistical analysis was performed using t-test with *** indicating $p < 0.001$, ** indicating $p < 0.01$ and * indicating $p < 0.05$. Error bars represent standard deviation.

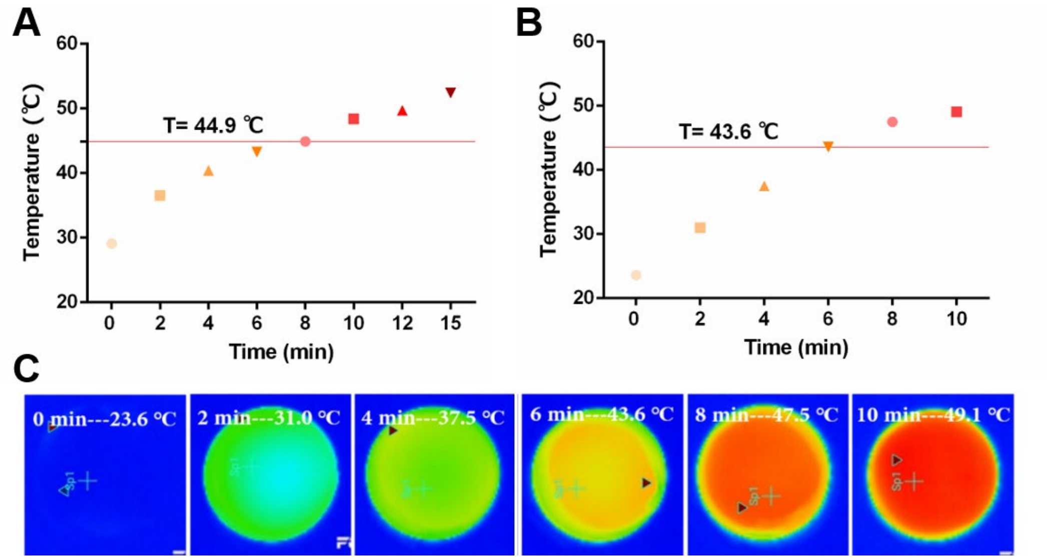

Figure S12. Temperature recorded during MTIT and MH groups in *in vitro* experiments. (A and **B)** Temperature changed within MH and MTIT groups with varied MH time respectively. **(C)** IR thermal images during MTIT treatment with varied MH time. U87 cells were incubated with RGD-IONs with iron concentration of 25 ug/mL for 24 h. Then, cells were treated with MTIT or MH respectively. The parameters of MH were 375 kHz, 10 min, while the parameters of MTIT were RMF 15 Hz, 15 min and AMF 375 kHz, 10min.

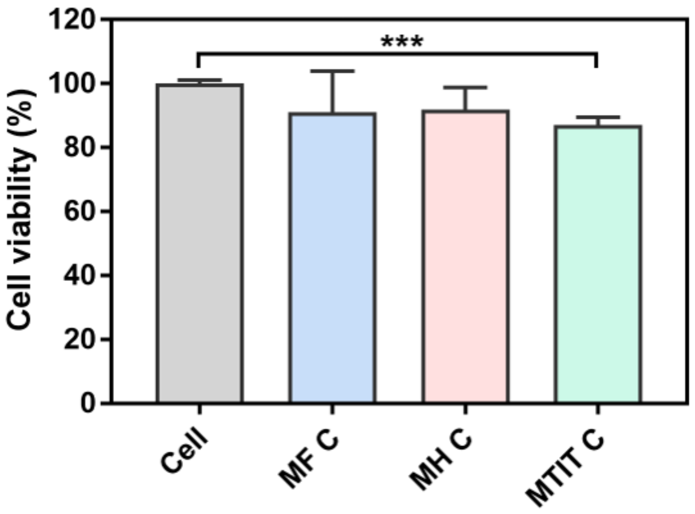

Figure S13. Cell viability of U87 cells in different treated groups. MF C represented magneto-mechanical force control, that cells without RGD-IONs were treated with MF with frequency of 15 Hz for 15 min. MH C represented magnetic hyperthermia control, that cells without RGD-IONs were treated with MH with frequency of 375 kHz for 6 min. MTIT C represented mechanical-thermal induction therapy control, that cells without RGD-IONs were treated with MF and MH orderly, and the parameters of the magnetic fields were same as above. The cell viability was measured post treatment 24 h via CCK-8 kit.

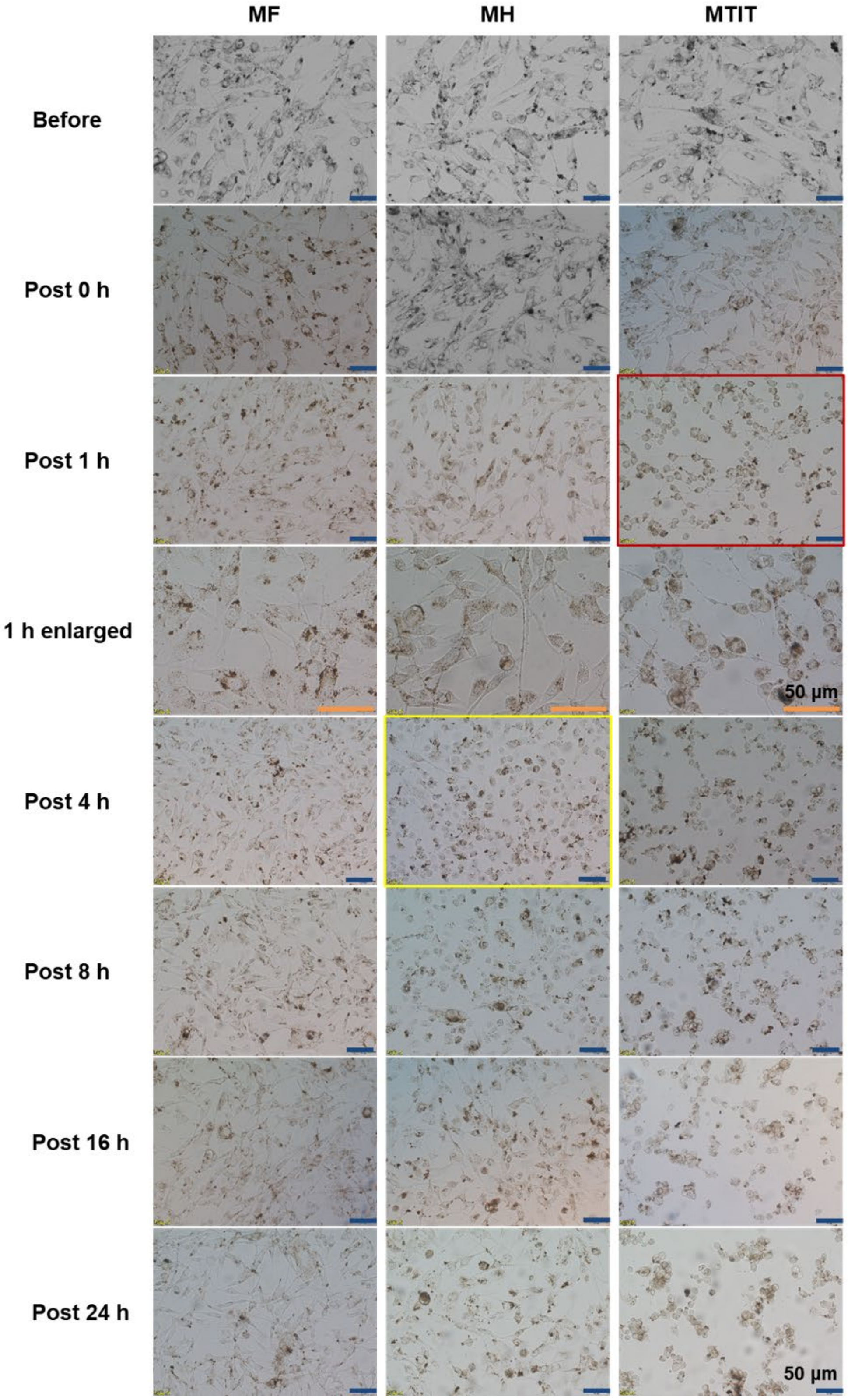

Figure S14. Cell structure evaluation. Cell morphology change of U87 cells in different treatment groups were captured post treatment 24 h. There was rounded shape of cells in MTIT groups after treatment 1 h, while rounded shape of cells were observed in MH groups after treatment 4 h.

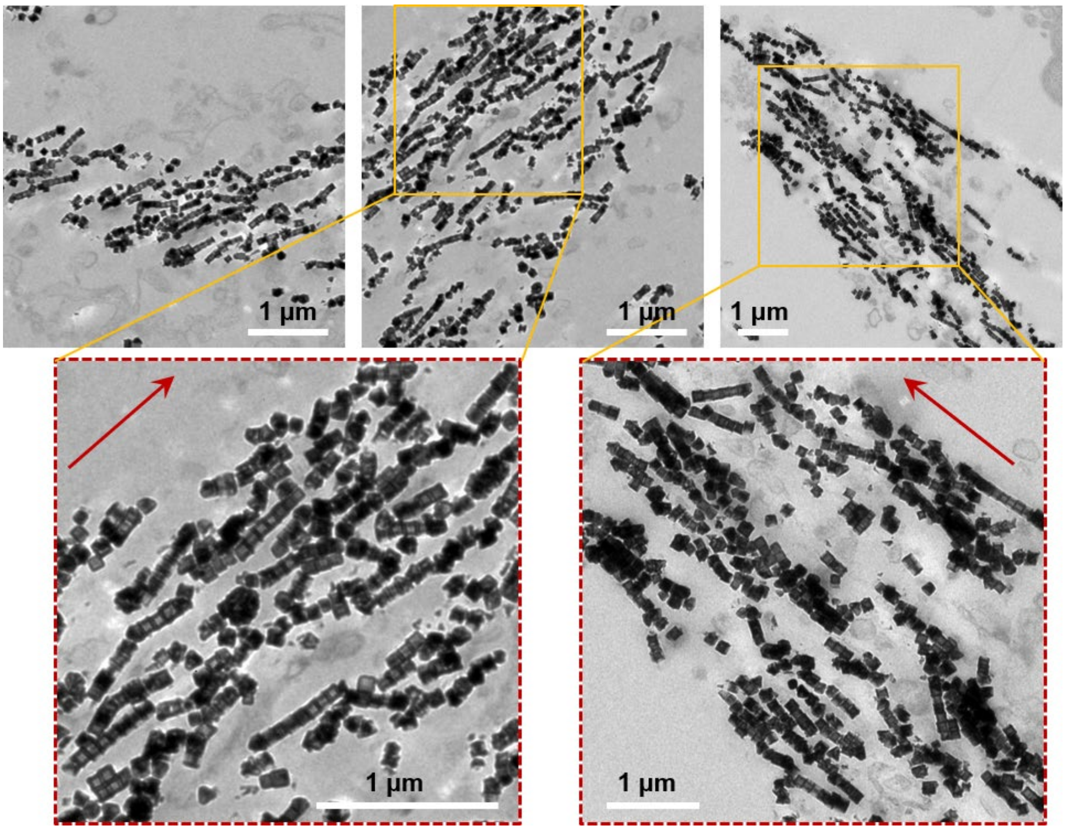

Figure S15. The alignment of RGD-IONs in MTIT group. The Bio-TEM sample was prepared after MTIT treatment. U87 cells were incubated with 25 μg/mL RGD-IONs for 24 h, followed by RMF (15 Hz, 40 mT, 15 min) and AMF (375 kHz, 12 kA/m, 6 min) (scale bar: 1 μm). Red arrows showed the directions of magnetic assemblies.

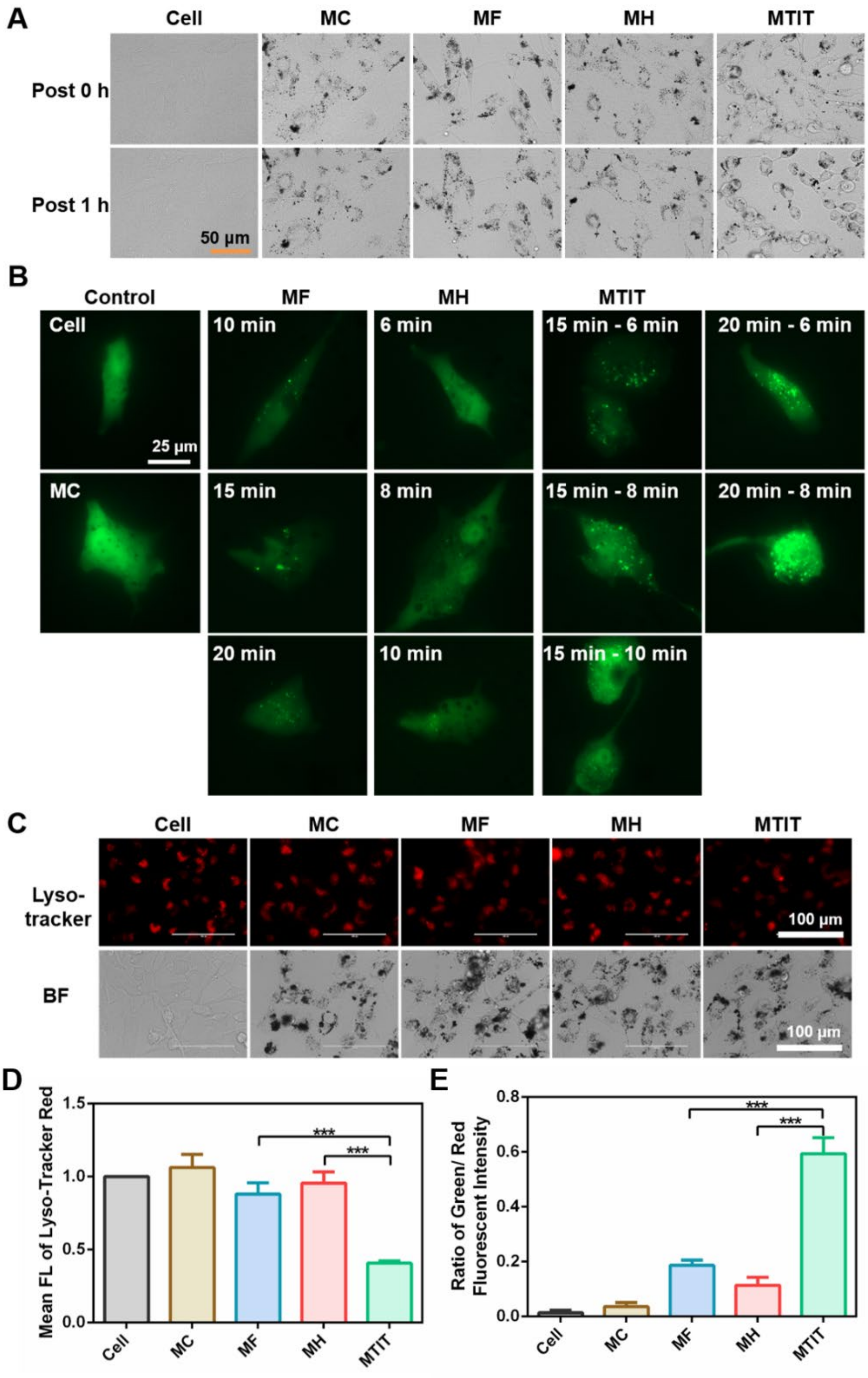

Figure S16. Subcellular structure changed characterization. (A) Cell morphology of U87 cells in different treatment groups were captured before and after treatment 1 h. **(B)** Lysosomal membrane permeabilization was evaluated by eGFP-Gal3 plasmid. The fluorescent spots represented the phenomenon of LMP. **(C)** Lyso-tracker red staining based on the acidic environment of lysosomes, was used to evaluate the change of LMP. **(D)** Mean fluorescence intensity of Lyso-Tracker Red quantified by ImageJ. **(E)** Quantitative analysis of JC-1 staining.

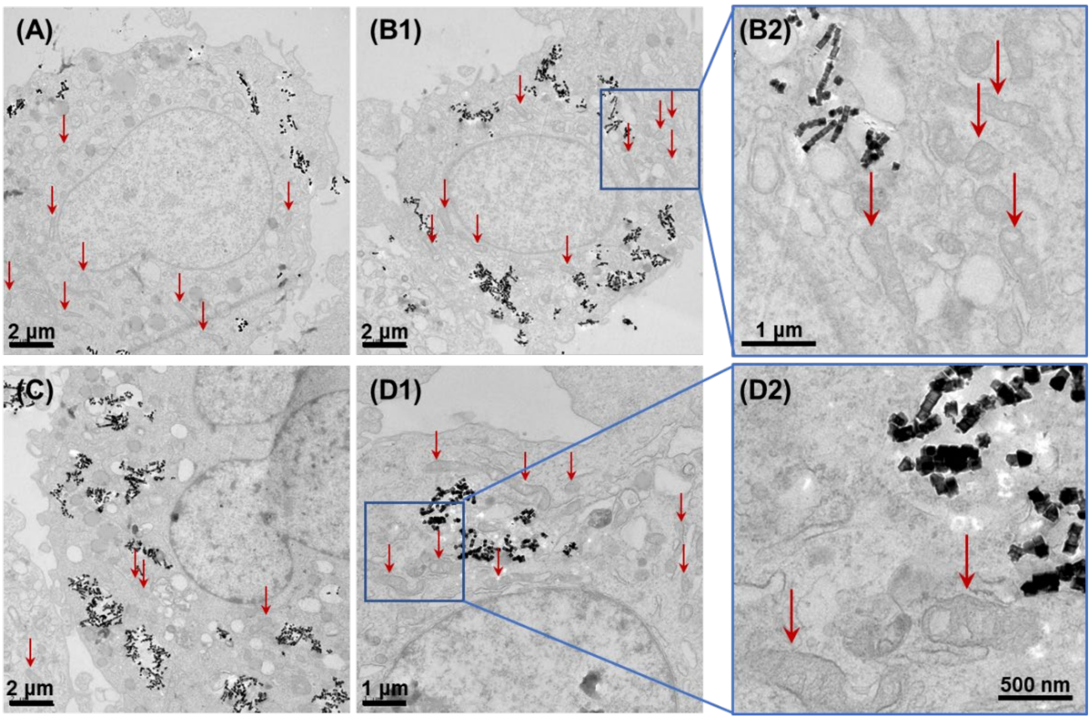

Figure S17. Colocalization of RGD-IONs and mitochondria based on Bio-TEM imaging. (A-D) The Bio-TEM images were captured after U87 cells incubated with 25 µg/mL RGD-IONs for 24 h. Red arrows represented the mitochondria. Nearly no RGD-IONs were directly bounded to or interacted with the mitochondria membrane.

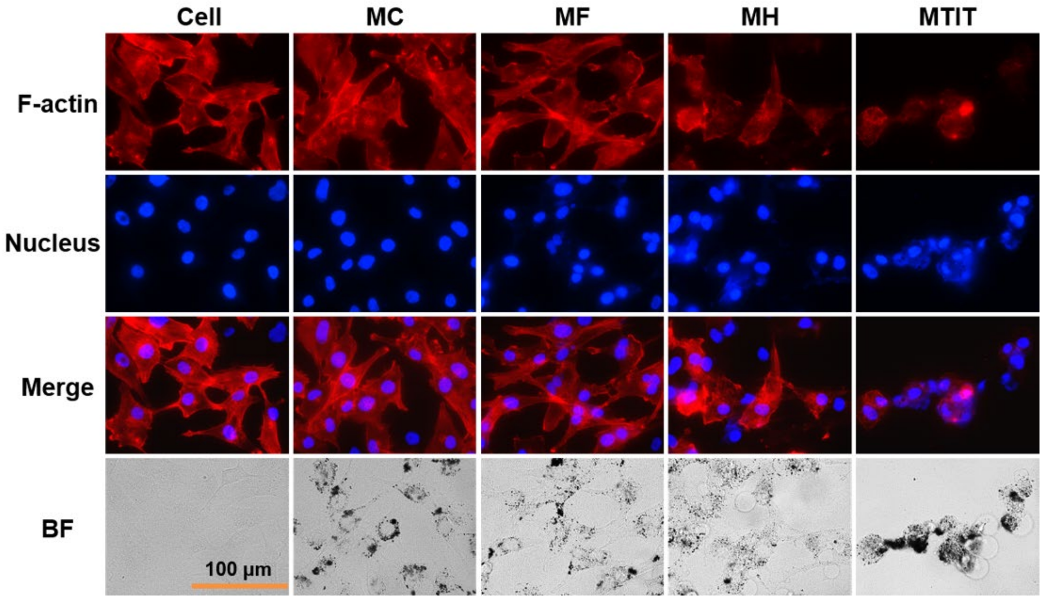

Figure S18. Cytoskeleton change of U87 cells after different treatment. Phalloidin was used to image the F-actin after treatment 24 h. The decreased integrity of F-actin demonstrated the obvious therapeutic effect of MTIT on cytoskeleton structure.

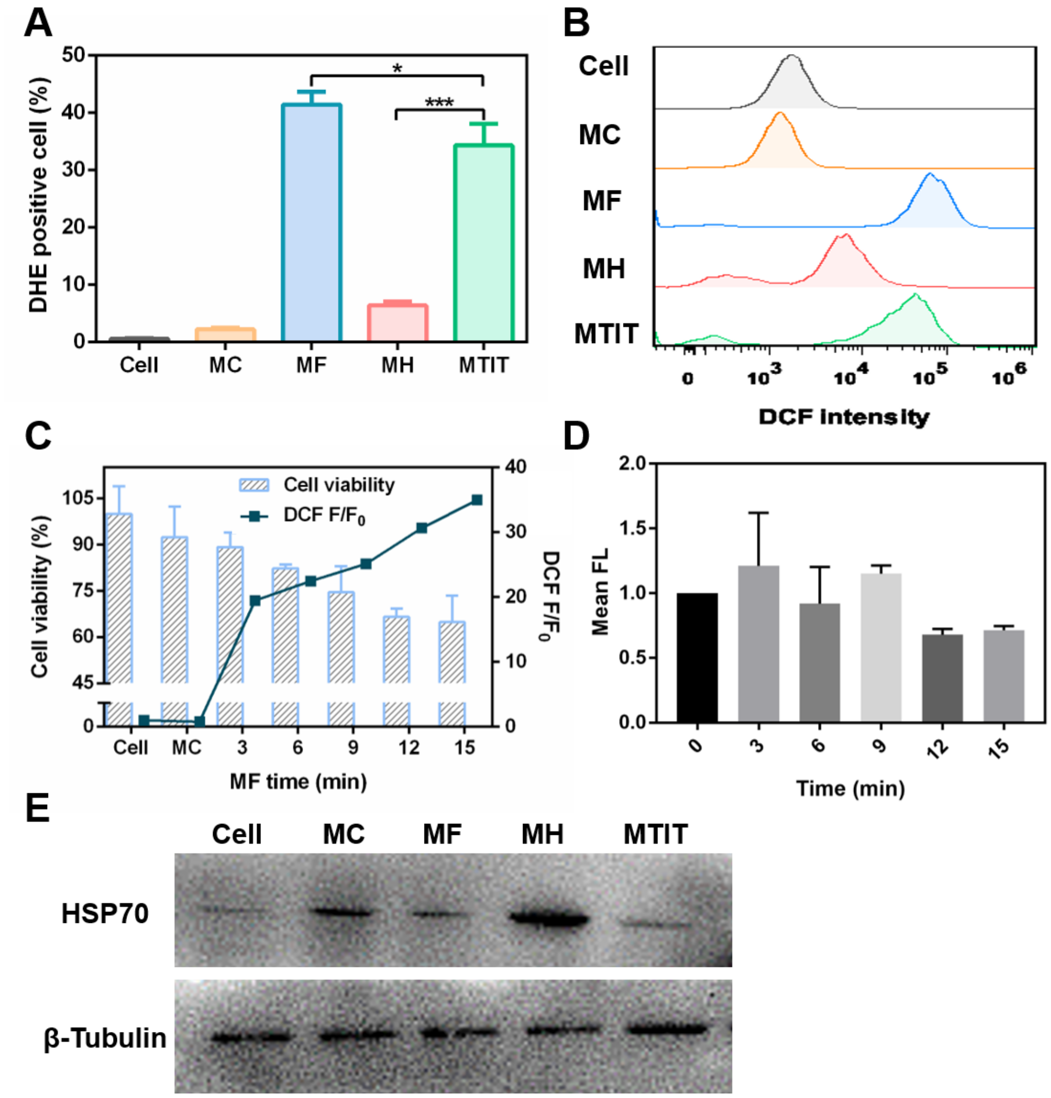

Figure S19. Biochemical signals detection, involving ROS and HSP70. (A) Quantitative analysis of DHE staining, demonstrating the high level of intracellular superoxide anion. **(B)** Intracellular ROS was investigated by DCFH-DA staining and quantified by flow cytometry. **(C)** Cell viability and ROS level with varied MF treated time in MF groups. U87 cells were coincubated with RGD-IONs with iron of 25 μg/mL, followed by the staining of DCFH-DA and MF treatments with different times. The cell viability was measured by CCK-8 kit post treatment 24 h. **(D)** The intracellular ROS level of U87 cells under RMF treatment for duration of 15 min without RGD-IONs. The cells were stained by DCFH-DA, followed by RMF treatment (15 Hz, 40 mT) with different treated times. Then the cells were collected for detection of ROS fluorescence via flow cytometry. **(E)** Western blot analysis showing the expression of HSP70.

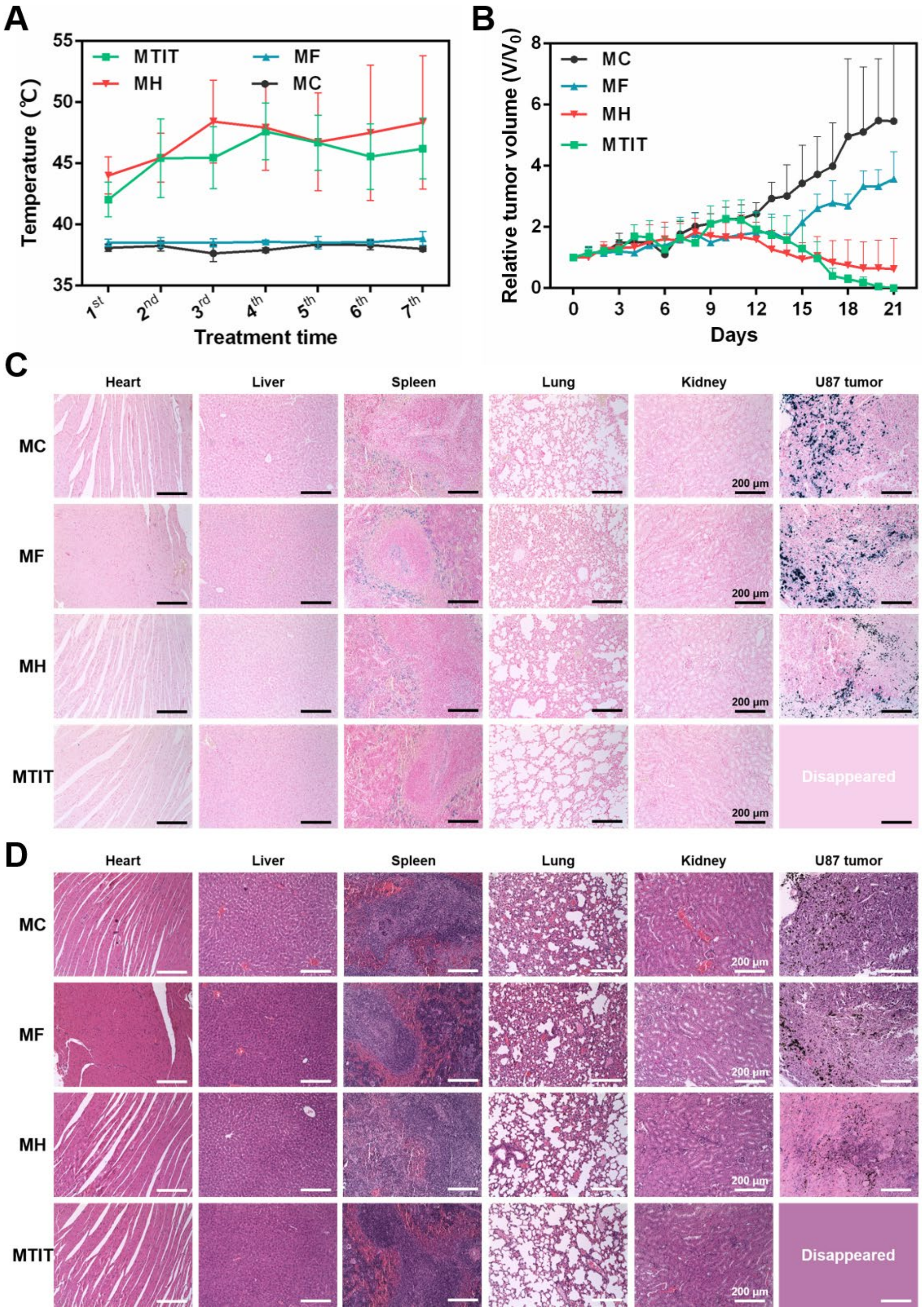

Figure S20. Animal experiments in U87-bearing mouse model. (A) Temperature recorded using infrared thermal camera during 7 times' treatment. (**B)** Relative tumor volume of mice in different treated groups. **(C)** Prussian blue staining of tumors and organs dissected from U87 - burdened mice. These U87 - burdened mice were intratumorally injected with RGD-MNPs with iron 5 mg/kg for three times at the day 0, 2 and 4. The treatment was performed for 7 times, every two days, from day 1 to day

13. At the day 21, the mice were dissected and the organs and tumors were collected for histological analysis (the scale bar: 200 μm). **(D)** H&E staining of organs dissected from U87 - burdened mice. No obvious damage was detected to normal tissues (the scale bar: 200 μm).

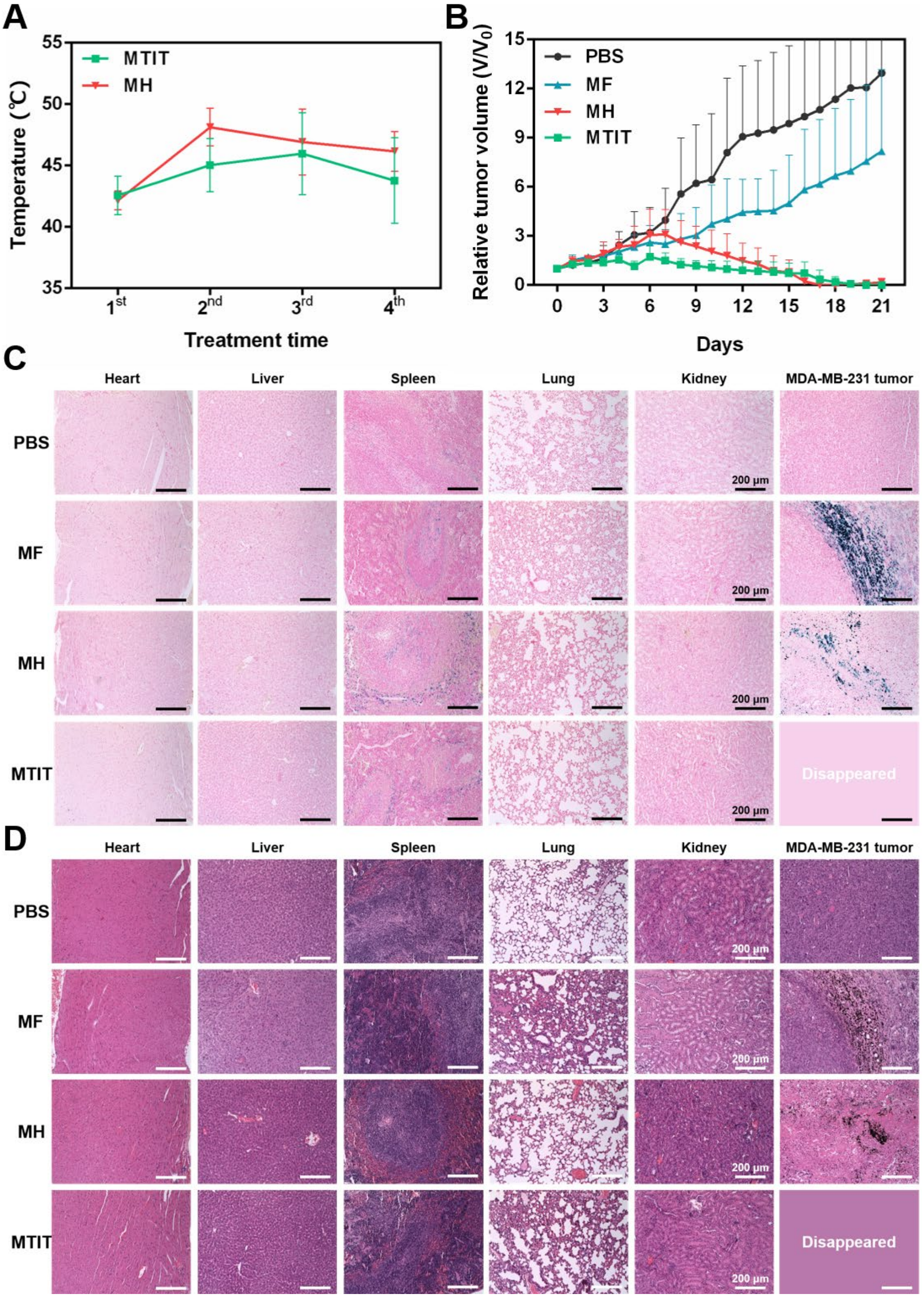

Figure S21. Animal experiments in triple negative breast cancer model. (A) Temperature recorded during 4 times' treatment in different treated groups. (**B)** Relative tumor volume of mice in different treated groups. **(C)** Prussian blue staining of tumors and organs dissected from MDA-MB-231 - burdened mice. These MDA-MB-231 - burdened mice were intratumorally injected with RGD-MNPs with iron 5 mg/kg for two times at the day 0 and 2. The treatment was performed for 4 times, at the day 1, 3,

5 and 7, respectively. At the day 21, the mice were dissected and the organs and tumors were collected for histological analysis (the scale bar: 200 μm). **(D)** H&E staining of organs dissected from MDA-MB-231-bearing mice. No obvious damage was detected to normal tissues (the scale bar: 200 μm).

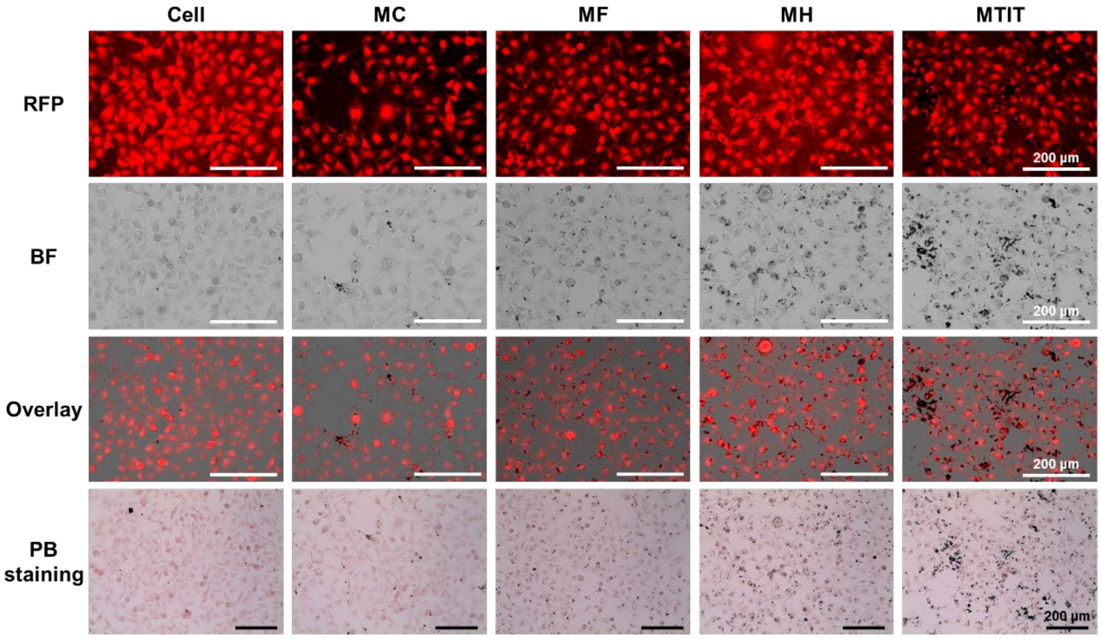

Figure S22. Reuptake of RGD-IONs in MDA-MB-231@RFP cells. RGD-IONs with iron concentration of 25 μg/mL were firstly internalized by MDA-MB-231 without RFP expression, subsequently, different treatments were performed to induce cell death. The parameters of RMF and AMF were same as before. Then, the cell fragments and leaked RGD-IONs were collected and co-incubated with the fresh MDA-MB-231@RFP cells. After coincubation of 24 h, the cells were stained by Prussian blue dyes. The images were captured to distinguish the RGD-IONs inside or outside of the cells (the scale bar: 200 μm).

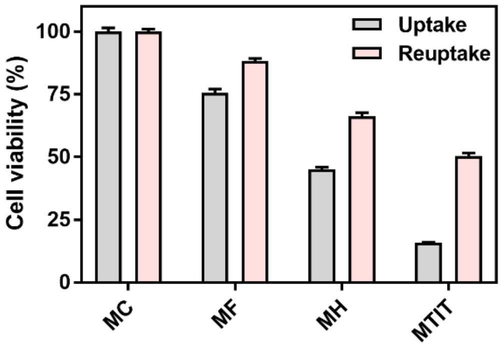

Figure S23. Compare of the cell viability of MTIT on MDA-MB-231 cells mediated with natural internalized and reinternalized RGD-IONs. The cell viability was measured by CCK-8 kit.

Table S1. Synergistic effect of MTIT with varied MF time.

MF (min)	MF cell viability (%)	MH (min)	MF-MH cell viability (%)	Q value
0	98.15±1.56	6	**62.33±1.00**	---
5	88.29±1.68	6	36.97±2.45	1.40
10	71.49±2.55	6	18.80±0.41	1.46
15	67.52±1.36	6	9.64±0.66	1.56
20	40.86±0.78	6	4.09±0.89	1.29
25	27.11±1.57	6	2.02±0.39	1.18
30	24.78±1.08	6	1.91±0.68	1.16

Table S2. Synergistic effect of MTIT with varied MH time.

MH (min)	MH cell viability (%)	MF (min)	MF-MH cell viability (%)	Q value
0	97.03±3.91	15	**67.52±1.36**	---
2	92.89±3.77	15	39.03±0.90	1.64
4	87.05±1.66	15	33.01±0.98	1.62
6	62.33±1.00	15	10.19±0.48	1.55
8	25.60±1.35	15	7.25±0.43	1.12
10	1.92±0.28	15	1.33±0.28	1.00
